# Changes in spatial clusters of cancer incidence and mortality over 15 years in South Korea: Implication to cancer control

**DOI:** 10.1002/cam4.6365

**Published:** 2023-07-25

**Authors:** Cham Thi Nguyen, Insang Song, Inkyung Jung, Yoon‐Jung Choi, Sun‐Young Kim

**Affiliations:** ^1^ Department of Cancer Control and Population Health, Graduate School of Cancer Science and Policy National Cancer Center Goyang‐si Republic of Korea; ^2^ Department of Geography University of Oregon Eugene Oregon USA; ^3^ Department of Biomedical Systems Informatics Yonsei University Seoul Republic of Korea; ^4^ Department of Cancer AI and Digital Health, Graduate School of Cancer Science and Policy National Cancer Center Goyang‐si Republic of Korea

**Keywords:** cluster analysis, incidence, mortality, neoplasms, prevention & control

## Abstract

**Background:**

The temporal investigation of high‐risk areas of cancer incidence and mortality can provide practical implications in cancer control. We aimed to investigate the changes in spatial clusters of incidence and mortality from 1999 through 2013 by major cancer types in South Korea.

**Methods:**

We applied flexible scan statistics to identify spatial clusters of cancer incidence and mortality by three 5‐year periods and seven major cancer types using the counts of new cases and deaths and population in 244 districts during 1999–2013. Then, we compared the changes across three periods in the locations of primary clusters of incidence and mortality by cancer types. To explore the determinants that possibly affect cancer cluster areas, we compared geographic characteristics between clustered and non‐clustered areas.

**Results:**

While incidence clusters for lung, stomach, and liver cancer remained in the same areas over 15 years, mortality clusters were relocated to the areas similar to those of incidence clusters. In contrast, colorectal, breast, cervical, and prostate cancer displayed consistently different locations of clusters over time, indicating the disappearance of existing clusters and the appearance of new clusters. Cluster areas tended to show higher portions of older population, unemployment, smoking, and cancer screening compared to non‐cluster areas particularly for mortality.

**Conclusions:**

Our findings of diverse patterns of changes in cancer incidence and mortality clusters over 15 years can indicate the degree of effectiveness in cancer prevention and treatment depending on the area and suggest the need for area‐specific applications of different cancer control programs.

## 1 INTRODUCTION

Cancer has remained the primary focus of public health globally for several decades, as cancer incidence and mortality have increased or been consistent despite the decline of most other chronic diseases.^1^, ^2^ In particular, new cases or deaths of cancer occurred disproportionately over space.^3^, ^4^ Spatial understanding of cancer mortality and incidence may provide solutions to reduce the high burden of cancer. To improve understanding, many studies of cancer have applied spatial analysis to search for high mortality or incidence areas, referred as spatial clusters, and subsequent studies identified characteristics that are associated with clustering patterns.^5^, ^6^ Cancer cluster investigation has been indicated as a useful statistical tool that allows the exploration of high event areas which are less likely to appear at random over space without designating a hypothesized association. In addition, it serves as the best fulfillment to respond to community concerns in public health practice, although the resulting putative clusters may not directly indicate associated etiologic agents.^7^ For example, studies of colorectal cancer in North America and Europe attempted to identify spatial clusters and related various determinants in neighboring physical and built environments, health behaviors, and socioeconomic conditions.^6^ A study of lung cancer mortality in China investigated spatial clusters over 1973–2013 and showed high smoking rate and advanced industrial development in identified clusters compared to other areas.^8^

Cancer incidence and mortality exhibit different pathways and progression as well as interplay between the two, which could result in similar or different locations of clusters. While cancer incidence directly reflects anatomical and histological characteristics and is directly affected by various risk factors,^9^ cancer mortality is considered as a function of incidence and survivorship.^10^ Specifically, new cancer cases could increase with the high prevalence of health‐related risk factors such as health behaviors, and environmental factors, or limited effectiveness of prevention programs to reduce these factors. On the contrary, cancer deaths could increase when medical intervention programs perform poorly, or fail to soften the impact of ongoing diseases or to avoid disabilities. The spatial variation in risk factors as well as prevention and intervention programs could result in spatial clusters of cancer incidence and mortality.

The identification of differences and similarities in clusters of cancer incidence and mortality over time can suggest future directions in evidence‐based interventions for cancer control. The geographic gap between incidence and mortality clusters could suggest the shortcomings in different prevention stages. The areas identified as an incidence cluster may indicate the limited effectiveness of health‐promoting and/or successful implementation of early‐detection programs, whereas a mortality cluster could mean the limitation in preventing severe symptoms and avoidable disabilities related to cancer mortality. Besides, the overlap of incidence and mortality clusters could suggest the difficulty in all stages of cancer prevention activities. Furthermore, cluster distribution in incidence and mortality could be consistent or vary over time and by cancer type, which provides the insights into the prioritizing areas of future interventions and the identification of geographic risk factors.^11^, ^12^

High burden of cancer and well‐established cancer registry in South Korea allow us to investigate cluster patterns of cancer incidence and mortality over an extended period. In South Korea, cancer has been a leading cause of death since 1983.^13^, ^14^ The South Korean government established the Korea Central Cancer Registry in 1980, and expanded to include the entire population in 1999 when nationwide cancer control programs began. District‐specific cancer incidence across 251–260 districts for 5‐year periods over 1999–2013 is available since 2016.^13^ District‐specific cancer mortality is also available since 1998 based on death certificate data. Using these spatially‐resolved cancer statistics data in South Korea, our study aimed to investigate spatial clusters in both cancer incidence and mortality by seven major cancer types in each of the three periods for 1999–2013, to investigate the changes in the cluster locations over the three periods, and to examine geographic characteristics to explain the differences and similarities between clustered and non‐clustered areas.

## 2 METHODS

### 2.1 Data

We obtained cancer counts, population, geographic characteristics, and district boundaries for each of the 251–260 districts in South Korea for 1999–2013 to apply spatial cluster analysis and to investigate cluster patterns with geographic characteristics. The total number of districts included to our cluster analysis was prune to 244 districts after we modified district boundaries to obtain the consistency over years. We downloaded the counts of new cancer cases and deaths by seven major cancer types for both sexes from the Korean Statistical Information Services (KOSIS) (https://kosis.kr/eng/). We selected the seven cancer types based on high incidence or mortality in South Korea. We defined the type of cancer by the International Classification of Diseases 10th revision (Table S1). District‐level cancer incidence is available as the aggregated counts for three 5‐year periods (1999–2003, 2004–2008, and 2009–2013) for confidentiality concerns given small numbers of specific cancer cases in some districts with relatively small population. For district‐level cancer death counts available annually, we aggregated to the same three 5‐year periods to those of incidence. We downloaded the mid‐year resident registration population and aggregated to the same three 5‐year periods.

We obtained shapefiles of district boundaries from the Statistical Geographical Information Service (https://sgis.kostat.go.kr/). South Korea was composed of seven Metropolitan Cities and nine Provinces during 1999–2011 (Figure S1). Each Metropolitan City or Province includes 1–48 districts with a total of 251–263 districts (median area = 391 [range = 3–1818] km^2^) for 1999–2013.^15^, ^16^ The number and boundary of districts have changed over years due to expansions, annexations, and partitions of district areas. For example, 246 districts for 1999–2000 decreased to 245 for 2001–2002 and increased to 251 for 2010–2013.^15^ To obtain the consistent boundaries to allow the application of cluster analysis and comparison of spatial clusters over years, we applied the district boundary in 2010 including 251 districts to the entire study period, and then excluded seven island districts. We modified the cancer counts and population in the districts over the other years with different boundaries from those in 2010. For a single district in 2010 combined from a few districts in previous years, we aggregated the counts from such districts. For the districts which were separate in 2010 from one district, we allocated the same cancer incidence and mortality rates to those multiple districts.

We used 11 geographic characteristics including demographic, socioeconomic, health‐related behavioral, and healthcare features (Table S2). These district‐specific geographic characteristics collected from the Community Health Survey (CHS) or computed by local governments are available in the KOSIS (https://kosis.kr/eng/). The CHS was designed as a nationwide, community‐based, and cross‐sectional survey that aims to produce comparable health statistics across districts.^17^ This questionnaire‐based survey, initiated in 2008, has collected a variety of health topics such as health behaviors and disease history annually from about 900 adults in each district. Given the data availability since 2008, we restricted our investigation of geographic characteristics to the final study period for 2009–2013 and did not consider for the other two early periods. For this analysis, we used the CHS data for a single year in 2010 that provides the most complete information. We used the local statistics data on the same year in 2010.

### 2.2 Statistical analysis

#### 2.2.1 Spatial cluster analysis

We identified spatial clusters using the scan method which is the most common approach of spatial cluster detection.^18^ The principle of spatial scan has been mentioned in previous literature.^19^, ^20^, ^21^ In brief, the spatial scan method relies on the null hypothesis where there is no cluster across study areas and searches through the set of areas as a candidate cluster using a scanning window with a predefined shape, size, and location. For each cluster candidate, the likelihood ratio is calculated as the ratio of likelihood inside the cluster candidate to all the areas outside of the cluster. A cluster candidate with the highest likelihood ratio is considered as the most likely cluster which is least likely to occur by chance and tested for statistical significance based on Monte Carlo simulation. As each combination of the shape, size, and location in a searching window could result in the variation of cluster detection, many efforts attempted to improve the accuracy and robustness of cluster detection and minimize the inconsistency in findings across different parameters. Previous studies of spatial scan methods commonly applied the scanning window with a circular or elliptical shape and a size of the median population over all study areas. However, real clusters may not have circular or elliptical shapes. In addition, some previous studies of cluster analyses showed that the circular scan method tended to include low‐event areas into clusters.^22^, ^23^, ^24^

In our cluster analysis, using the counts of new cases and deaths as well as population across 244 districts, we applied flexible scan statistics along with Poisson model by three different periods and seven cancer types.^24^ Flexible scan statistics employ the cluster candidate created by connecting adjacent areas for each given area (i.e., district in our study) using the default maximum window size as 50% of the total number of areas.^25^ This flexibility overcomes the limitations of the traditional spatial scan method that relies on a searching window with a circular or elliptical shape and population‐based size. Moreover, the restricted likelihood ratio method allows the inclusion of areas to a cluster only when such areas give the significantly large number of cases. This additional restriction can help avoid the false‐positive findings. Since flexible scan considers connections of each area for cluster detection, the detection is computationally demanding. To reduce the load, we applied the maximum size of a scanning window as 15% (37 districts), instead of 50%, as a previous simulation study showed that the number of areas in a significant cluster is unlikely to exceed 10%–15% of the total number of areas.^22^, ^26^ Although multiple clusters could be reported, we only presented the primary cluster, as the most likely cluster which showed the highest likelihood ratio. We did not consider the secondary clusters, which provide significantly large likelihood ratios less than primary cluster, because their significance is assessed by ignoring the existence of the primary cluster and this ignorance could lead to a loss in statistical power.^27^

We performed two sensitivity analyses to examine the robustness of our findings. First, we decreased the window size to 10% and 5% of the total number of districts and compared the cluster locations to those from our primary analysis using 15%. Additionally, we carried out the same cluster analysis by two sexes separately.

#### 2.2.2 Relationship of clusters with geographic characteristics

To examine geographic characteristics that possibly distinguish clustered districts from non‐clustered districts, we compared 11 health‐related characteristics by seven cancer types during the last period for 2009–2013. We compared each characteristic between cluster versus non‐cluster districts by incidence and mortality. Because some clusters are composed of small numbers of districts less than five, we included all statistically significant clusters including primary and secondary clusters in order to retain sufficient numbers of districts for comparison. All statistical analyses were implemented in R version 4.1.3 with the “rflexscan” package for cluster analysis (R Development Core Team https://www.r‐project.org/).

## 3 RESULTS

### 3.1 Spatial distribution of cancer incidence and mortality

**FIGURE 1:**
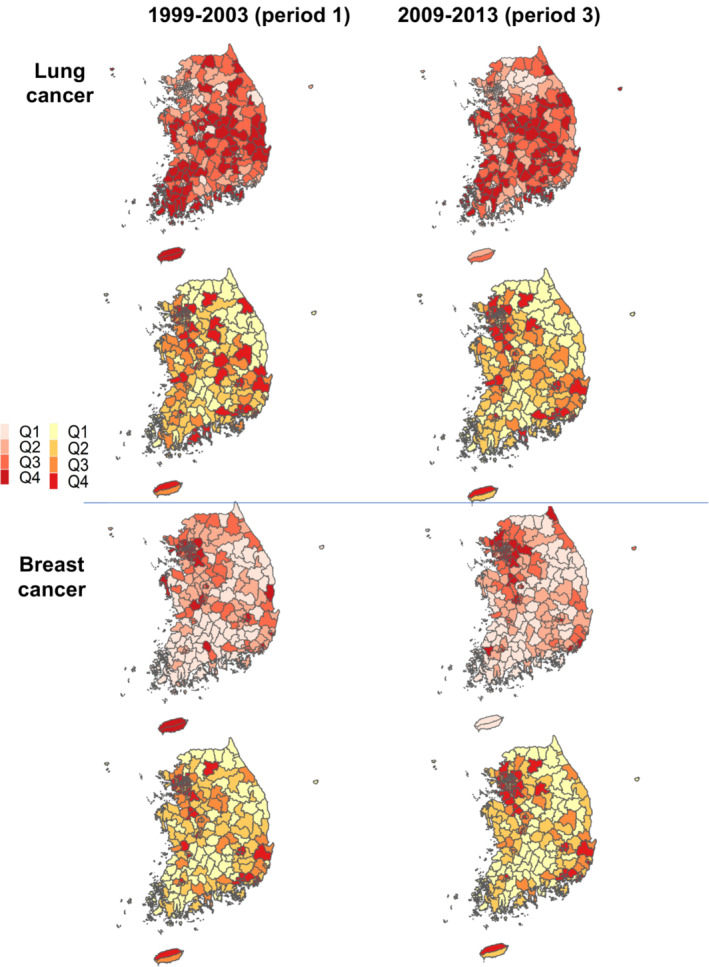
Maps of crude incidence (above) and mortality (below) for lung and breast cancer across 244 districts in the first and last 5‐year periods over 1999–2013 in South Korea.

During the 15‐year period from 1999 through 2013, the numbers of district‐specific new cancer cases and cancer deaths in South Korea increased for all cancer types except cervical cancer for incidence and stomach cancer for mortality (Table S3 and Figure S2). Figure 1 shows the spatial distribution of incidence and mortality rates as the counts of new cases and deaths relative to the population for two cancers, lung and breast cancer. Both showed temporally consistent patterns of substantial increase in incidence and mortality rates, while spatially different patterns were observed in high incidence or mortality areas. For instance, lung cancer showed consistently high incidence and mortality in the southern region including mostly rural areas over time. In contrast, breast cancer displayed high incidence in Metropolitan Cities including Seoul. While stomach and liver cancers had similar patterns to those of lung cancer, sex‐specific cancers such as cervical and prostate cancers showed different patterns (Figure S3).

### 3.2 Cluster locations of incidence and mortality over 15 years across cancer types

**FIGURE 2:**
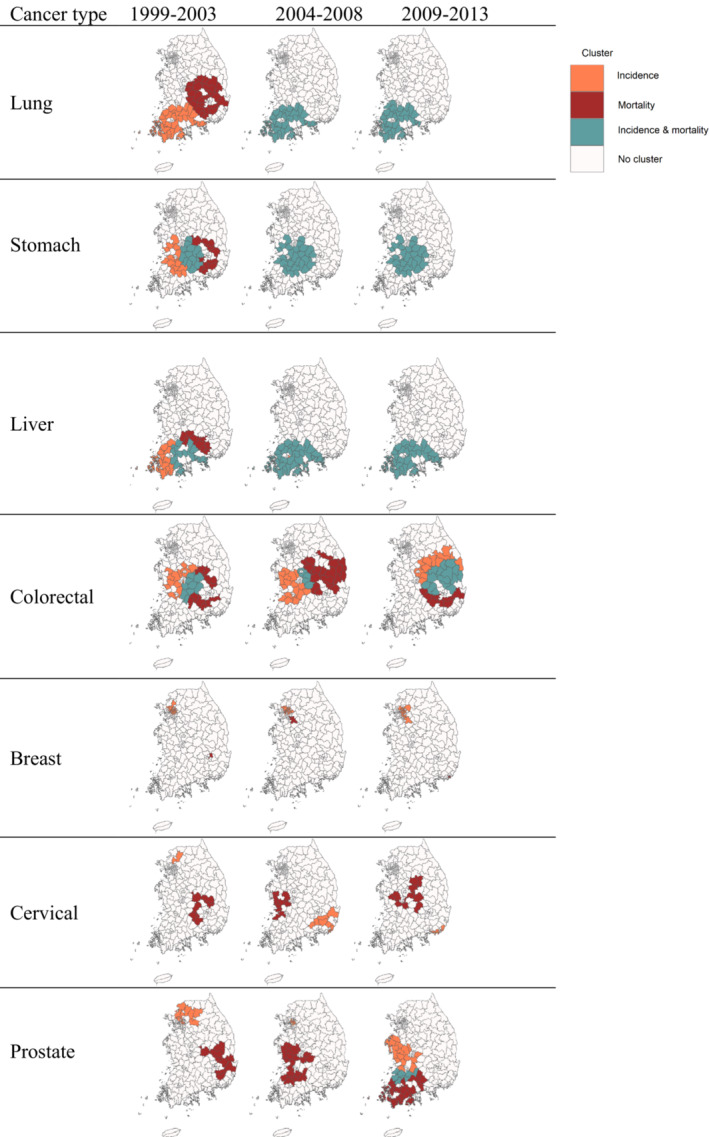
Maps of cluster areas for incidence and mortality by seven cancer types and three time periods for 1999–2013 in South Korea.

Locations of primary clusters were similar or different depending on the incidence and mortality as well as cancer type in the earliest period, period 1, for 1999–2003 (Figure 2). The clusters of lung and liver cancer incidence were found in the southwestern region, while stomach and colorectal cancer incidence showed the clusters in the central region. For reproductive cancers, the cluster of breast cancer incidence was seen in two Metropolitan Cities including Seoul and Daegu, whereas the clusters of cervical and prostate cancer incidence were located in the northern areas of Seoul. Mortality clusters were located in the neighboring regions of incidence clusters for lung, stomach, and liver cancer. However, reproductive cancer clusters were found in distant locations from incidence clusters.

**FIGURE 3:**
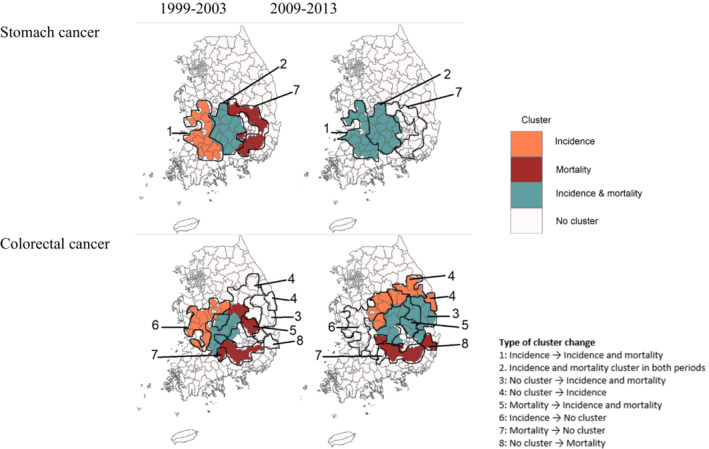
Eight types of changes in cancer incidence and mortality clusters between the period 1 (1999–2003) and period 3 (2009–2013) by stomach and colorectal cancer in South Korea.

The changes of cluster locations in the later periods for 2004–2013 from the period 1 also varied by cancer outcomes and sites (Figures 2 and 3). For lung, stomach, and liver cancer, primary clusters of incidence seen in the southwestern region in the period 1 was also found in the same region in the periods 2 and 3, shown as the Types 1 and 2 of cluster change in Figures 3 and Figure S4. Moreover, mortality clusters in the eastern region in the period 1 did not exist any longer in the period 3 (Type 7) and remained in the same region as completely overlapped clusters with incidence (Type 2). Different from these cancers, colorectal cancer showed the change of cluster locations in both incidence and mortality. The incidence cluster found in the western region for the periods 1 and 2 was not displayed in the period 3 (Type 6) and new cluster areas appeared in the east (Types 4 and 5). The new cluster area is also found for mortality in the southeastern area (Type 8), while some mortality cluster areas remained and overlapped with the new incidence cluster (Type 7). Although it is not common, a few areas without any clusters in the period 1 became identified as the cluster for both incidence and mortality (Type 3) The change of cluster locations was most notable in reproductive cancer. Breast cancer showed the change of mortality cluster locations in the period 2 and the disappearance in the period 3, while incidence clusters were found in similar or new areas over time. Cervical and prostate cancer showed changes in both incidence and mortality clusters over time. These findings were generally consistent when we reduced the window size to 10% of the total number of districts in our sensitivity analysis (Figure S5). When we stratified by sex, the pattern was also consistent between males and females but with different locations of the clusters (Figure S6).

### 3.3 Relationships of cancer clusters with geographic characteristics

In our investigation of geographic characteristics in the period 3, some characteristics showed the differences between cluster and non‐cluster areas of cancer incidence and mortality. Incidence clusters in most cancer types were found in the areas with higher proportion of older adults, smokers, and cancer screening compared to non‐cluster areas (Table S4). In addition, the cluster areas were characterized by lower socioeconomic status including lower educational attainment, higher unemployment, and lower gross regional domestic product. As one exception, the cluster areas of breast cancer incidence were composed of urban, relatively young, and highly educated population. Clusters of cancer mortality generally showed similar but larger differences compared to those of incidence (Table S5). For instance, unemployment rate, proportion of current smoking, and participation in cancer screening were much higher in mortality cluster areas than non‐cluster areas compared to incidence. It is worth noting that breast cancer incidence and mortality showed different patterns of the geographic characteristics in cluster versus non‐cluster areas. The average current and secondhand smoking rates were similar between cluster and non‐cluster areas in incidence, while it was higher in mortality cluster areas. Although incidence cluster areas had lower average rate of breast cancer screening than that in non‐cluster areas, the opposite is found in mortality.

## 4 DISCUSSION

Our study applied a flexible approach to identify spatial clusters of cancer incidence and mortality, as potential high‐risk areas, across seven major cancer types and to explore the changes over 15 years from 1999 through 2013. Incidence and mortality clusters showed different patterns in changes over time depending on the cancer type. For lung, stomach, and liver cancer, incidence clusters remained in the same areas, while mortality clusters disappeared or moved to similar areas to those of incidence. In contrast, colorectal, female breast, cervical, and prostate cancer generally displayed different locations of incidence and mortality clusters over time, indicating the presence of new clusters and the absence of existing clusters. The clustered areas were commonly characterized by older population, higher smoking rate, and higher participation rate in cancer screening compared to non‐clustered areas, particularly for mortality.

This study adds important findings of the changing patterns of potential high‐risk areas in cancer incidence and mortality jointly over time, which can improve our understanding of the relationships with cancer control programs and provide practical guidance for future interventions. While many studies investigated high‐risk areas of cancer using cluster analysis, some studies focused on temporal changes.^6^, ^11^, ^28^, ^29^ However, there were few studies that looked at both incidence and mortality collectively with the consideration of multiple cancer types. Along with increasing cancer incidence and mortality worldwide over a few decades, there have been tremendous efforts of cancer control including various prevention and treatment interventions which affect the spatial patterns of incidence and mortality sequentially rather than simultaneously. As South Korea established the nationwide cancer control programs in late 1990s, cancer survival rate of 42.9% in 1993–1995 dramatically increased to 70.7% in 2015–2019^30^, ^31^ and cancer screening rates increased from 1.2%–4.2% to 33.6%–73.6% depending on the cancer type.^32^ Our investigation of incidence and mortality clusters over the following 15 years since this establishment can highlight the advances and challenges resulting from the expansion of cancer control efforts.

As an attempt to provide practical guidance based on our findings, we summarized in Figure S7 the eight types of cluster changes between periods 1 and 3, which are visualized in Figures 3 and S4, and provided their possible explanations related to cancer control. For example, the Type 1 indicates the areas found as the cancer incidence cluster in the period 1 and turned into the cluster of incidence as well as morality in the period 3, possibly suggesting the lagged effect of incidence increase on mortality reduction. Increased incidence might have resulted in mortality increase in a short time while effective treatment interventions were not yet implemented. Our finding of high proportion of cancer screening in incidence clusters and much higher proportion in mortality clusters may also mean this lag effect where effective cancer screening increases early detection but mortality decrease does not follow yet. The change from no cluster to incidence cluster in the Type 4 may suggest the increase in incidence resulting from active implementation of cancer screening or prolonged effect of early‐life exposure.^33^, ^34^ The identification of these areas with less effective prevention and/or treatment environments can provide guidance to future cancer control. In contrast, the disappearance of incidence and mortality clusters may mean effective preventive interventions and improved treatment advances as the success of cancer control.

Our findings showed similar change patterns of cluster locations for one set of cancer types but different patterns for the other: lung, stomach, and liver cancer versus colorectal, breast, prostate, and cervical cancer. Over 15 years, lung, stomach, and liver cancer tended to show incidence clusters consistently in the same areas with some overlap for mortality clusters. However, colorectal, female breast, cervical, and prostate cancer generally displayed changes of cluster locations for both incidence and mortality clusters with little overlap. These two classes of cancer types align with preventable and treatable cancer mortality conceptualized by the Organization for Economic Co‐operation and Development (OECD). Preventable death is defined as causes of death that can be avoided by implementing effective public health and primary preventive interventions before the stage of disease onsets, while treatable death is characterized as avoidable deaths through timely and effective health care interventions including secondary prevention and treatment after the onset of disease.^35^ The OECD applied these concepts to cancer and classified cancer types into two classes: lung, stomach, and liver cancer as preventable mortality and colorectal, breast, cervical, and prostate cancer as treatable mortality.^36^ Our findings of non‐overlapping clusters such as high incidence but low mortality in the same area for cancers related to treatable mortality could be derived by their slow progress and favorable prognosis. These cancer types, in particular, generally showed increasing or consistent patterns in age‐standardized incidence and mortality rates over time in most middle‐ and high‐income countries, even though most other cancer sites displayed decreasing trends.^30^, ^35^ Furthermore, their survivorship has considerably improved compared to cancers in preventable mortality.^37^ These recent patterns of cancers in treatable mortality suggest further attentions in cancer control.

Geographic characteristics partly explain the distinctive properties in cluster areas compared to non‐cluster areas. The cluster areas of incidence in 2009–2013 were characterized by older population, higher unemployment, higher current smoking rate, and higher cancer screening rate than those of non‐cluster areas in all but breast cancer. These patterns were found even stronger in mortality. Breast cancer was an exception in that cancer screening rate in incidence clusters was higher than non‐cluster areas, while the lower rate was found in mortality clusters. These findings are consistent to previous literature. For example, lung cancer clusters were found in the areas with high smoking rate in China and low socioeconomic status in Pennsylvania, United states, while colorectal cancer clusters were detected in the areas with high screening rates in North Carolina, United states.^38^, ^39^ Another U.S. study of breast cancer incidence and mortality across more than 3000 counties showed that the counties with low socioeconomic status had low rates of breast cancer screening possibly resulting in low incidence and high mortality.^36^

We applied flexible scan statistics that allow us to overcome the limitations of traditional scan methods.^24^ Cancer could be developed over extended time periods related to built and/or physical environments and various geographic characteristics including socioeconomic conditions and public health interventions shared across neighboring administrative units, such as districts. Thus, the application of a window that identifies the cluster based on such commonly‐affected areas could be a more favored approach compared to fixed shapes such as circles or elliptics used in traditional approaches. Moreover, the large variation of the district size with small districts in urban areas and large districts in rural areas of South Korea could make it difficult to apply a predefined shape to detect a cluster.

Our study includes several limitations that promote further studies. First, we investigated the patterns of changing clusters from 1999 through 2013, but this 15‐year period may not be sufficient to observe the complete pattern of changes. The decreasing trend of nationwide cancer incidence and mortality rates beginning in the middle of 2010s, as interpreted as the achievement of nationwide cancer control programs, also suggests the need of the extended investigation. Future studies should re‐examine the changes by adding the updated cancer data for the latest periods. Second, we did not account for age structure in our cluster detection. As cancer incidence and mortality tend to be high in the areas with high proportion of order adults, cluster analyses for cancer could have used age‐standardized rate or have adjusted for age when aiming to identify new clusters or new risk factors after excluding the impact of age structure. However, our study focused on the temporal changes in the locations of spatial clusters and their potential relationships with cancer control actions which also include older adult population. Third, our investigation of geographic characteristics focused on the difference between clustered and non‐clustered areas in the period 3 rather than the change of cluster locations across three periods, because of data limitation. The addition of geographic characteristics in addition to cancer incidence and mortality data in the latest periods can allow us to investigate the relationships of changes in geographic characteristics and changes in cancer clusters. Finally, the local characteristic description of cluster versus non‐cluster areas may not confirm the causal relationship between health risk factors and cluster areas. Further studies could investigate whether cluster locations change depending on the adjustment and apply a novel approach to investigate the causal association with geographic risk factors.^40^

## 5 CONCLUSIONS

Our study investigated the temporal changes in the spatial clusters of incidence and mortality over 15 years since nationwide cancer control programs began in South Korea and found various types of sequential changes depending on the cancer type. The change including persistence, relocation, removal, or introduction of clusters may suggest enhanced or limited effectiveness of cancer prevention and/or treatment interventions and provide practical guidance to future cancer control programs for such area.

## AUTHOR CONTRIBUTIONS

**Cham Thi Nguyen:** Conceptualization (lead); data curation (lead); formal analysis (lead); investigation (lead); methodology (lead); validation (lead); visualization (lead); writing – original draft (lead). **Insang Song:** Methodology (supporting); validation (supporting); writing – review and editing (supporting). **Inkyung Jung:** Methodology (supporting); writing – review and editing (supporting). **Yoon‐Jung Choi:** Investigation (supporting); writing – review and editing (supporting). **Sun‐Young Kim:** Conceptualization (lead); formal analysis (supporting); funding acquisition (lead); investigation (supporting); methodology (supporting); resources (supporting); supervision (lead); writing – review and editing (lead).

## FUNDING STATEMENT

This work was supported by the National Research Foundation of Korea (grant number: 2022R1A2C2009971) and the National Cancer Center of Korea (NCC‐2110570, NCC‐2310220).

## CONFLICT OF INTEREST STATEMENT

The authors have no conflicts of interest to declare.

## ETHICAL APPROVAL

Ethics approval and informed consent not applicable.

## Supporting information


Data S1:

## Data Availability

All the data including district‐specific cancer incidence, mortality, and geographic characteristics used in our study are available in the Korean Statistical Information Services (https://kosis.kr/eng/).

## References

[1] Bray F , Laversanne M , Weiderpass E , Soerjomataram I . The ever‐increasing importance of cancer as a leading cause of premature death worldwide. Cancer. 2021;127(16):3029‐3030. 34086348 10.1002/cncr.33587

[2] Mahase E . Cancer overtakes CVD to become leading cause of death in high income countries. BMJ. 2019;366:l5368. 31481521 10.1136/bmj.l5368

[3] Torre LA , Siegel RL , Ward EM , Jemal A . Global cancer incidence and mortality rates and trends—an update. Cancer Epidemiol Biomarkers Prev. 2016;25(1):16‐27. 26667886 10.1158/1055-9965.EPI-15-0578

[4] Thun MJ , DeLancey JO , Center MM , Jemal A , Ward EM . The global burden of cancer: priorities for prevention. Carcinogenesis. 2010;31(1):100‐110. 19934210 10.1093/carcin/bgp263 PMC2802672

[5] Goodman M , Naiman JS , Goodman D , LaKind JS . Cancer clusters in the USA: what do the last twenty years of state and federal investigations tell us? Crit Rev Toxicol. 2012;42(6):474‐490. 22519802 10.3109/10408444.2012.675315 PMC3408895

[6] Soffian SSS , Nawi AM , Hod R , Chan HK , Hassan MRA . Area‐level determinants in colorectal cancer spatial clustering studies: a systematic review. Int J Environ Res Public Health. 2021;18(19):10486. 34639786 10.3390/ijerph181910486 PMC8508304

[7] CDC . Guidelines for Investigating Clusters of Health Events. 1990 Accessed July 27, 1990 https://www.cdc.gov/mmwr/preview/mmwrhtml/00001797.htm 2117247

[8] Shen X , Wang L , Zhu L . Spatial analysis of regional factors and lung cancer mortality in China, 1973‐2013. Cancer Epidemiol Biomarkers. 2017;26(4):569‐577. 10.1158/1055-9965.EPI-16-0922 28223434

[9] Jemal A , Parkin DM , Bray F . Patterns of Cancer Incidence, Mortality, and Survival. Cancer Epidemiology and Prevention [Internet]. Oxford University Press; 2017. doi:10.1093/oso/9780190238667.003.0008

[10] Horner RD , Chirikos TN . Survivorship differences in geographical comparisons of cancer mortality: an urban‐rural analysis. Int J Epidemiol. 1987;16(2):184‐189. 3610445 10.1093/ije/16.2.184

[11] Amin RW , Fritsch BA , Retzloff JE . Spatial clusters of breast cancer mortality and incidence in the contiguous USA: 2000‐2014. J Gen Intern Med. 2019;34(3):412‐419. 30652275 10.1007/s11606-018-4824-9 PMC6420677

[12] Jang J , Yoo D‐S , Chun BC . Spatial epidemiologic analysis of the liver cancer and gallbladder cancer incidence and its determinants in South Korea. BMC Public Health. 2021;21(1):2090. 34774036 10.1186/s12889-021-12184-8 PMC8590754

[13] Won Y‐J , Jung K‐W , Oh C‐M , et al. Geographical variations and trends in major cancer incidences throughout Korea during 1999‐2013. Cancer Res Treat. 2018;50(4):1281‐1293. 29334607 10.4143/crt.2017.411 PMC6192921

[14] Shin H‐Y , Lee J‐Y , Song J , et al. Cause‐of‐death statistics in the Republic of Korea, 2014. J Korean Med Assoc. 2016;59:221.

[15] Korea S . Statistical Geographic Information Service. https://sgis.kostat.go.kr/view/index.jsp

[16] Safety MoIa . Cities and Provinces in Korea. 2021 https://www.mois.go.kr/eng/sub/a03/citiesProvinces/screen.do

[17] Kang YW , Ko YS , Kim YJ , et al. Korea community health survey data profiles. Osong Public Health Res Perspect. 2015;6(3):211‐217. 26430619 10.1016/j.phrp.2015.05.003 PMC4551141

[18] Roquette R , Painho M , Nunes B . Spatial epidemiology of cancer: a review of data sources, methods and risk factors. Geospat Health. 2017;12(1):504. 28555468 10.4081/gh.2017.504

[19] Kulldorff M . A spatial scan statistic. Commun Statist‐Theory Meth. 1997;26(6):1481‐1496.

[20] Bernoulli KM . Discrete Poisson and continuous Poisson models: a spatial scan statistic. Communications in Statistics: theory and methods. Commun Statist‐Theory Meth. 1997;26:1481‐1496.

[21] Kulldorff M , Huang L , Pickle L , Duczmal L . An elliptic spatial scan statistic. Stat Med. 2006;25(22):3929‐3943. 16435334 10.1002/sim.2490

[22] Tango T , Takahashi K . A flexibly shaped spatial scan statistic for detecting clusters. Int J Health Geogr. 2005;4(1):11. 15904524 10.1186/1476-072X-4-11 PMC1173134

[23] Tango T . Spatial scan statistics can be dangerous. Stat Methods Med Res. 2021;30(1):75‐86. 33595399 10.1177/0962280220930562

[24] Tango T , Takahashi K . A flexible spatial scan statistic with a restricted likelihood ratio for detecting disease clusters. Stat Med. 2012;31(30):4207‐4218. 22807146 10.1002/sim.5478

[25] Otani T , Takahashi K . Flexible scan statistics for detecting spatial disease clusters: the rflexscan R package. J Stat Softw. 2021;99(13):1‐29.

[26] Ribeiro SHR , Costa MA . Optimal selection of the spatial scan parameters for cluster detection: a simulation study. Spat Spatiotemporal Epidemiol. 2012;3(2):107‐120. 22682437 10.1016/j.sste.2012.04.004

[27] Zhang Z , Assunção R , Kulldorff M . Spatial scan statistics adjusted for multiple clusters. J Probab Stat. 2010;2010:642379.

[28] Leiser CL , Taddie M , Hemmert R , et al. Spatial clusters of cancer incidence: analyzing 1940 census data linked to 1966‐2017 cancer records. Cancer Causes Control. 2020;31(7):609‐615. 32323050 10.1007/s10552-020-01302-3 PMC7574665

[29] Amin RW , Stafford B , Guttmann RP . A spatial study of bladder cancer mortality and incidence in the contiguous US: 2000‐2014. Sci Total Environ. 2019;670:806‐813. 30921714 10.1016/j.scitotenv.2019.03.290

[30] Kang MJ , Won YJ , Lee JJ , et al. Cancer statistics in Korea: incidence, mortality, survival, and prevalence in 2019. Cancer Res Treat. 2022;54(2):330‐344. 35313102 10.4143/crt.2022.128 PMC9016309

[31] Jung KW , Yim SH , Kong HJ , et al. Cancer survival in Korea 1993‐2002: a population‐based study. J Korean Med Sci. 2007;22 Suppl(Suppl):S5‐s10. 17923755 10.3346/jkms.2007.22.S.S5 PMC2694370

[32] Suh M , Choi KS , Park B , et al. Trends in cancer screening rates among Korean men and women: results of the Korean National Cancer Screening Survey, 2004‐2013. Cancer Res Treat. 2016;48(1):1‐10. 25943324 10.4143/crt.2014.204 PMC4720068

[33] Levin TR , Corley DA , Jensen CD , et al. Effects of organized colorectal cancer screening on cancer incidence and mortality in a large community‐based population. Gastroenterology. 2018;155(5):1383‐91.e5. 30031768 10.1053/j.gastro.2018.07.017 PMC6240353

[34] Steinmaus C , Ferreccio C , Acevedo J , et al. Increased lung and bladder cancer incidence in adults after In utero and early‐life arsenic exposure. Cancer Epidemiol Biomarkers Prev. 2014;23(8):1529‐1538. 24859871 10.1158/1055-9965.EPI-14-0059 PMC4344186

[35] Sung H , Ferlay J , Siegel RL , et al. Global cancer statistics 2020: GLOBOCAN estimates of incidence and mortality worldwide for 36 cancers in 185 countries. CA Cancer J Clin. 2021;71(3):209‐249. 33538338 10.3322/caac.21660

[36] Development OfEC‐oa . Avoidable mortality: OECD/Eurostat lists of preventable and treatable causes of death. Accessed January 2022 2022.

[37] Jung K‐W , Park S , Kong H‐J , et al. Cancer statistics in Korea: incidence, mortality, survival, and prevalence in 2009. Cancer Res Treat. 2012;44(1):11‐24. 22500156 10.4143/crt.2012.44.1.11 PMC3322196

[38] Zhu Y , McKeon TP , Tam V , Vachani A , Penning TM , Hwang WT . Geographic differences in lung cancer incidence: a study of a major metropolitan area within southeastern Pennsylvania. Int J Environ Res Public Health. 2020;17(24):9498. 33352953 10.3390/ijerph17249498 PMC7767044

[39] Kuo TM , Meyer AM , Baggett CD , Olshan AF . Examining determinants of geographic variation in colorectal cancer mortality in North Carolina: a spatial analysis approach. Cancer Epidemiol. 2019;59:8‐14. 30640041 10.1016/j.canep.2019.01.002

[40] Nethery RC , Yang Y , Brown AJ , Dominici F . A causal inference framework for cancer cluster investigations using publicly available data. J R Stat Soc Ser A Stat Soc. 2020;183(3):1253‐1272. 10.1111/rssa.12567 PMC8276584 34262243

