# Supplementary material for: Changes in spatial clusters of cancer incidence and mortality over 15 years in South Korea: Implication to cancer control
Source: Cancer Med. 2023 Jul 25;12(16):17418–27. doi: 10.1002/cam4.6365 (PMC10501259; doi:10.1002/cam4.6365)
Supplement: Supplementary file 1 — Data S1: [file CAM4-12-17418-s001.docx]

**SUPPLEMENTAL MATERIAL**

**Changes in spatial clusters of cancer incidence and mortality over 15 years in South Korea: implication to cancer control**

Nguyen Thi Cham, Insang Song, Inkyung Jung, Yoon-Jung Choi, Sun-Young Kim

**List of supplemental tables**

[Table S1. Definition of cancer types according to the International Classification of Diseases 10^th^ revision (ICD-10) code 4](#_Toc131005588)

[Table S2. Eleven district-specific characteristics for 2009-2013 in South Korea 4](#_Toc131005589)

[Table S3. Summary statistics of the numbers of new cancer cases and deaths across 244 districts by seven cancer types and three time periods for 1999-2013 in South Korea 5](#_Toc131005590)

[Table S4. Averages of 11 district-specific geographic characteristics between cluster versus non-cluster areas of cancer incidence by seven cancer types during 2009-2013 6](#_Toc131005591)

[Table S5. Averages of 11 district-specific geographic characteristics between cluster versus non-cluster areas of cancer mortality by seven cancer types during 2009-2013 7](#_Toc131005592)

**List of supplemental figures**

[Figure S 1. Map of administrative boundaries in South Korea, 2010 8](#_Toc131005593)

[Figure S 2. Box plots of the numbers of new cases (A) and deaths (B) across 244 districts by three time periods and seven cancer types for 1999-2013 in South Korea 9](#_Toc131005594)

[Figure S 3. Maps of crude incidence (above) and mortality (below) for seven cancers across 244 districts and three periods for 1999-2013 in South Korea 12](#_Toc131005595)

[Figure S 4. Change of incidence and mortality clusters over 15 years from 1999 through 2013 and possible explanation based on the nationwide cancer control program that has expanded since establishment in 1999 in South Korea 13](#_Toc131005596)

[Figure S 5. Maps of primary clusters of cancer incidence and mortality by different scanning window size across 244 districts and three periods for 1999-2013 in South Korea 15](#_Toc131005597)

[Figure S 6. Maps of primary clusters of cancer incidence and mortality by males and females across 244 districts and three periods for 1999-2013 in South Korea 16](#_Toc131005598)

[Figure S 7. Eight types of changes in cancer incidence and mortality clusters between the period 1 (1999-2003) and period 3 (2009-2013) by other cancer types in South Korea 18](#_Toc131005599)

Table S1. Definition of cancer types according to the International Classification of Diseases 10^th^ revision (ICD-10) code

| Cancer type | Description | ICD-10 code |
| --- | --- | --- |
| Lung cancer | Malignant neoplasms of trachea, bronchus, and lung | C33-C34 |
| Stomach cancer | Malignant neoplasms of stomach | C16 |
| Liver cancer | Malignant neoplasms of liver and intrahepatic bile ducts | C22 |
| Colorectal cancer | Malignant neoplasms of colon, rectum, and anus | C18-C21 |
| Breast cancer | Malignant neoplasm of breast | C50 |
| Cervical cancer | Malignant neoplasm of cervix uteri, uterus | C53-C55 |
| Prostate cancer | Malignant neoplasm of prostate | C61 |

Table S2. Eleven district-specific characteristics for 2009-2013 in South Korea

| Category | Characteristic | Description | Source* | Year |
| --- | --- | --- | --- | --- |
| Population demographic | Elderly | Proportion of population aged 65 and above to the total population in each district | CHS | 2010 |
|  | Urban | Proportion of population in urban areas | CHS |  |
| Socioeconomic status | Gross Regional Domestic Proportion (GRDP) | The aggregate of gross value added of all resident producer units in the district (USD) | LS |  |
|  | High education | Proportion of people who graduated from high schools and above | CHS |  |
|  | Unemployment | Proportion of the unemployed to the economically active population | CHS |  |
| Health behaviors | Current smoking | Percentage of current smokers (“daily” or “occasionally” smokers) who have smoked at least 5 packs (100 cigarettes) in their lifetime | CHS |  |
|  | Secondhand smoking | Percentage of people who have smelled smoke from someone else's cigarettes indoors at home in the past week | CHS |  |
| Screening | Any cancer | Proportion of actual examinees to eligible people for any cancer screening | CHS |  |
|  | Specific cancer | Proportion of actual examinees to eligible people for cancer screening of the corresponding cancer | CHS |  |
| Healthcare infrastructure | Hospital bed | Number of hospital beds per 1000 people | LS | 2011 |
|  | Medical personnel | Number of medical personnel per 1000 people | LS | 2006 |

*CHS: Community Health Survey; LS: Local statistics

Table S3. Summary statistics of the numbers of new cancer cases and deaths across 244 districts by seven cancer types and three time periods for 1999-2013 in South Korea

| Cancer types | Period | Incidence | | | | Mortality | | | |
| --- | --- | --- | --- | --- | --- | --- | --- | --- | --- |
|  |  | Min | Mean | Max | SD* | Min | Mean | Max | SD |
| Lung | 1999-2003 | 0 | 610 | 1,938 | 339 | 13 | 210 | 668 | 119 |
|  | 2004-2008 | 51 | 780 | 3,052 | 482 | 20 | 312 | 1312 | 194 |
|  | 2009-2013 | 92 | 976 | 5,536 | 703 | 28 | 360 | 1842 | 251 |
| Stomach | 1999-2003 | 0 | 970 | 3,306 | 585 | 15 | 200 | 639 | 117 |
|  | 2004-2008 | 100 | 1,162 | 4,400 | 776 | 27 | 240 | 1049 | 155 |
|  | 2009-2013 | 92 | 1,390 | 8,232 | 1,097 | 17 | 220 | 1124 | 159 |
| Liver | 1999-2003 | 0 | 589 | 2,158 | 371 | 24 | 182 | 669 | 115 |
|  | 2004-2008 | 58 | 668 | 3,020 | 460 | 16 | 246 | 1023 | 170 |
|  | 2009-2013 | 71 | 732 | 3,784 | 552 | 17 | 253 | 1320 | 192 |
| Colorectal | 1999-2003 | 0 | 524 | 2,210 | 386 | 5 | 84 | 332 | 58 |
|  | 2004-2008 | 64 | 883 | 3,796 | 683 | 10 | 143 | 739 | 107 |
|  | 2009-2013 | 94 | 1,244 | 7,836 | 1,035 | 10 | 178 | 918 | 139 |
| Breast | 1999-2003 | 0 | 315 | 1,486 | 298 | 0 | 22 | 113 | 19 |
|  | 2004-2008 | 26 | 501 | 2,822 | 496 | 1 | 37 | 271 | 35 |
|  | 2009-2013 | 38 | 729 | 6,147 | 777 | 2 | 46 | 354 | 46 |
| Cervical | 1999-2003 | 0 | 194 | 796 | 154 | 0 | 24 | 103 | 18 |
|  | 2004-2008 | 0 | 179 | 892 | 148 | 1 | 29 | 128 | 22 |
|  | 2009-2013 | 0 | 172 | 988 | 154 | 0 | 29 | 172 | 25 |
| Prostate | 1999-2003 | 0 | 81 | 388 | 65 | 0 | 12 | 52 | 8 |
|  | 2004-2008 | 0 | 211 | 1,284 | 190 | 2 | 23 | 140 | 18 |
|  | 2009-2013 | 0 | 393 | 3,748 | 368 | 3 | 32 | 178 | 24 |

*SD: standard deviation

Table S4. Averages of 11 district-specific geographic characteristics between cluster versus non-cluster areas of cancer incidence by seven cancer types during 2009-2013

| Geographical characteristics ^(1)^ | | Lung | | Stomach | | | Liver | | | Colorectal | | Breast | | | Cervical | | Prostate | |
| --- | --- | --- | --- | --- | --- | --- | --- | --- | --- | --- | --- | --- | --- | --- | --- | --- | --- | --- |
| Cluster | | Yes  (N ^(2)^ = 129) | No  (115) | Yes  (131) | No  (113) | Yes  (131) | | No  (113) | Yes  (134) | | No  (110) | Yes  (50) | No  (194) | Yes  (69) | | No  (175) | Yes  (115) | No  (129) |
| Population demographic | Elderly | **19.5** ^(3)^ | **19** | **7.6** | **7.5** | **19** | | **7.6** | **19.2** | | **7** | 8.9 | 14.9 | **16** | | **12.8** | **19.4** | **8.7** |
|  | Urban | 53.2 | 56.2 | 71.5 | 73.8 | 56.2 | | 71.5 | 59.9 | | 67.4 | **83.2** | **58.2** | 67.9 | | 61.4 | 58.6 | 67.5 |
| Socio-economic status | GRDP | 1,321 | 1,434 | 3,583 | 3,778 | 1,434 | | 3,583 | 1,861 | | 3,120 | 4,221 | 1,968 | 1,787 | | 2,688 | 1,823 | 2,971 |
|  | High education | 9.4 | 10.1 | 15.7 | 16.1 | 10.1 | | 15.7 | 11.3 | | 14.4 | **21.0** | **10.5** | 11.3 | | 13.3 | 12.3 | 13.0 |
|  | Unemployment | **1.6** | **1.4** | **1.2** | **1.1** | 1.4 | | 1.2 | **1.4** | | **1.2** | 0.5 | 1.5 | 1.3 | | 1.3 | 1.5 | 1.1 |
| Health behaviors | Current smoking | **23.9** | **23.6** | **20.8** | **21.1** | **23.6** | | **20.8** | **24.1** | | 20.2 | 20.3 | 22.9 | **23.6** | | **21.8** | **23.4** | **21.4** |
|  | Secondhand smoking | 23.9 | 24.8 | 27.1 | 28.4 | 24.8 | | 27.1 | 24.8 | | 27.0 | 26.9 | 25.6 | 25.7 | | 25.9 | 23.9 | 27.5 |
| Screening | Any cancer | **48.1** | **47.6** | **37.6** | **37.7** | **47.6** | | **37.6** | **48.3** | | **36.4** | 38.8 | 44.0 | **45.2** | | **42.0** | **48.9** | **48.6** |
|  | Specific cancer | - | 41.9 | **33.4** | **33.1** | **41.9** | | **33.4** | **31.6** | | **24.1** | 39.8 | 47.3 | **36.1** | | **30.9** | - | - |
| Healthcare infrastructure | Hospital bed | **0.68** | **0.70** | **0.50** | **0.55** | **0.70** | | **0.50** | **0.70** | | **0.50** | 0.74 | 0.59 | **0.78** | | **0.06** | 0.67 | 0.57 |
|  | Medical personnel | 1.30 | 1.35 | 1.16 | 1.20 | 1.35 | | 1.16 | 1.40 | | 1.10 | **1.98** | **1.08** | 1.60 | | 1.31 | 1.38 | 1.15 |
| (1) Elderly: proportion of population aged 65 and above to the total population in each district; Urban: proportion of population in urban areas; GRDP: Gross regional domestic product (USD); High education: proportion of people who graduated from high schools and above; Unemployment: proportion of the unemployed to the economically active population; current smoking: percentage of current smokers (“daily” or “occasionally” smokers) who have smoked at least 5 packs (100 cigarettes) in their lifetime; Secondhand smoking: percentage of people who have smelled smoke from someone else's cigarettes indoors at home in the past week; any cancer screening: proportion of actual examinees to eligible people for any cancer screening; specific cancer screening: proportion of actual examinees to eligible people for cancer screening of the corresponding cancer; hospital bed: number of hospital beds per 1000 people; medical personnel: number of medical personnel per 1000 people;  (2) Number of districts  (3) Bold numbers with statistically significant difference between cluster and non-cluster areas | | | | | | | | | | | | | | | | | | |

Table S5. Averages of 11 district-specific geographic characteristics between cluster versus non-cluster areas of cancer mortality by seven cancer types during 2009-2013

| Geographical characteristics ^(1)^ | | Lung | | Stomach | | Liver | | Colorectal | | Breast | | Cervical | | Prostate | | |
| --- | --- | --- | --- | --- | --- | --- | --- | --- | --- | --- | --- | --- | --- | --- | --- | --- |
| Cluster | | Yes  (N^2)^ = 123) | No  (121) | Yes  (124) | No  (120) | Yes  (123) | No  (131) | Yes  (109) | No  (135) | Yes  (6) | No  (138) | Yes  (32) | No  (212) | Yes  (66) | No  (178) |  |
| Population demographic | Elderly | **19.8^(3)^** | **7.2** | **19.9** | **7.4** | **19.5** | **7.9** | **20.8** | **8.0** | 12.7 | 13.8 | **17.2** | **13.2** | **23.2** | **10.2** |  |
|  | Urban | 53.6 | 73.6 | 52.6 | 74.3 | 54.7 | 72.1 | 55.1 | 69.9 | **100.0** | **62.0** | 67.3 | 62.7 | 45.1 | 70.0 |  |
| Socio-economic status | GRDP | 1,315 | 3,619 | 1,215 | 3,685 | 1,248 | 3,629 | 1,164 | 3,450 | 2,157 | 2,436 | 1,278 | 2,603 | 888 | 3,000 |  |
|  | High education | 9.5 | 16.1 | 9.6 | 15.8 | 9.7 | 15.7 | 9.6 | 15.2 | 18.3 | 12.5 | 11.1 | 12.9 | 8.5 | 14.2 |  |
|  | Unemployment | **1.6** | **0.9** | **1.6** | **0.97** | **1.5** | **1.1** | **1.6** | **1.1** | 0 | 1.3 | 1.4 | 1.3 | **1.7** | **1.1** |  |
| Health behaviors | Current smoking | **24.2** | **20.3** | **23.8** | **20.9** | **23.5** | **21.2** | **23.9** | **21.1** | **24.7** | **22.3** | **24.8** | **21.9** | **23.4** | **21.9** |  |
|  | Secondhand smoking | 24.6 | 27.1 | **23.7** | 20.9 | 24.0 | 27.7 | 22.8 | 28.3 | **30.5** | **25.7** | 26.2 | 25.8 | 21.1 | 27.6 |  |
| Screening | Any cancer | **48.9** | **36.6** | **48.5** | **37.1** | **47.8** | **37.9** | **49.2** | **37.9** | 42.8 | 42.9 | **47.1** | **42.3** | **51.2** | **39.9** |  |
|  | Specific cancer | **-** | - | 47.4 | 32.8 | **42.1** | **33.7** | **30.9** | **26.0** | **54.4** | **45.5** | **31.6** | **37.6** | - | - |  |
| Healthcare infrastructure | Hospital bed | **1.38** | **1.01** | **1.37** | **1.03** | **1.40** | **0.98** | **1.43** | **1.02** | 2.20 | 1.20 | 1.40 | 1.17 | 1.35 | 1.14 |  |
|  | Medical personnel | 2.60 | 2.20 | **2.60** | **2.20** | **2.70** | **2.10** | **2.73** | **2.10** | **3.00** | 2.30 | 2.80 | 2.30 | 2.43 | 2.37 |  |
| 1) Elderly: proportion of population aged 65 and above to the total population in each district; Urban: proportion of population in urban areas; GRDP: Gross regional domestic product (USD); High education: proportion of people who graduated from high schools and above; Unemployment: proportion of the unemployed to the economically active population; current smoking: percentage of current smokers (“daily” or “occasionally” smokers) who have smoked at least 5 packs (100 cigarettes) in their lifetime; Secondhand smoking: percentage of people who have smelled smoke from someone else's cigarettes indoors at home in the past week; any cancer screening: proportion of actual examinees to eligible people for any cancer screening; specific cancer screening: proportion of actual examinees to eligible people for cancer screening of the corresponding cancer; hospital bed: number of hospital beds per 1000 people; medical personnel: number of medical personnel per 1000 people;  (2) Number of districts  (3) Bold numbers with statistically significant difference between cluster and non-cluster areas | | | | | | | | | | | | | | | | |


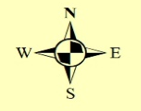

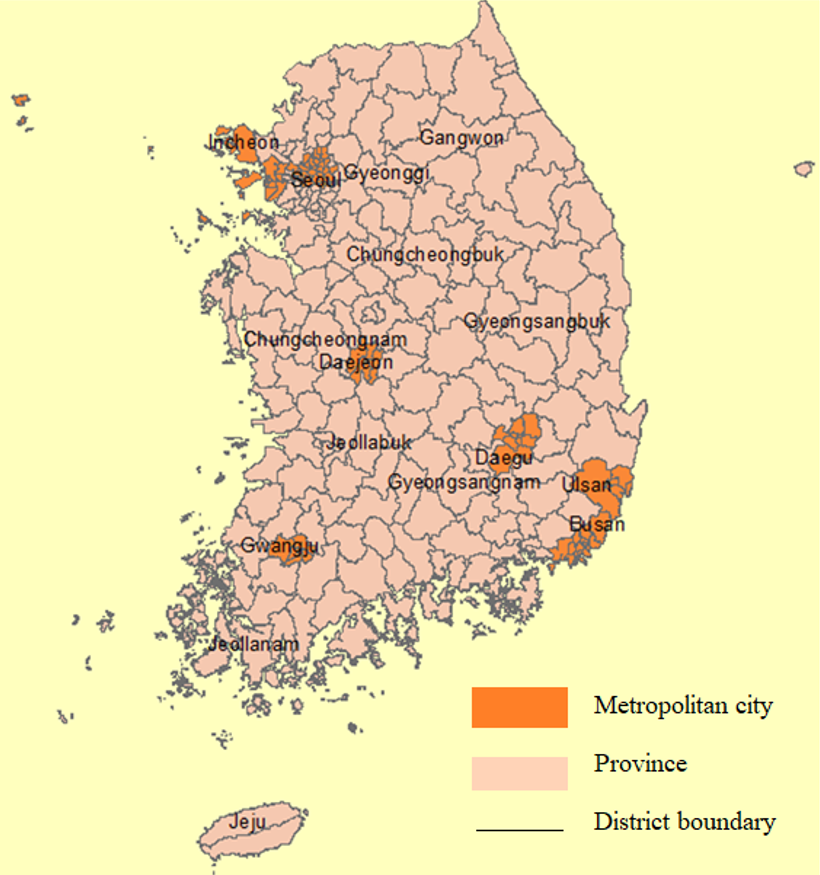


Figure S 1. Map of administrative boundaries in South Korea, 2010

(A) Incidence


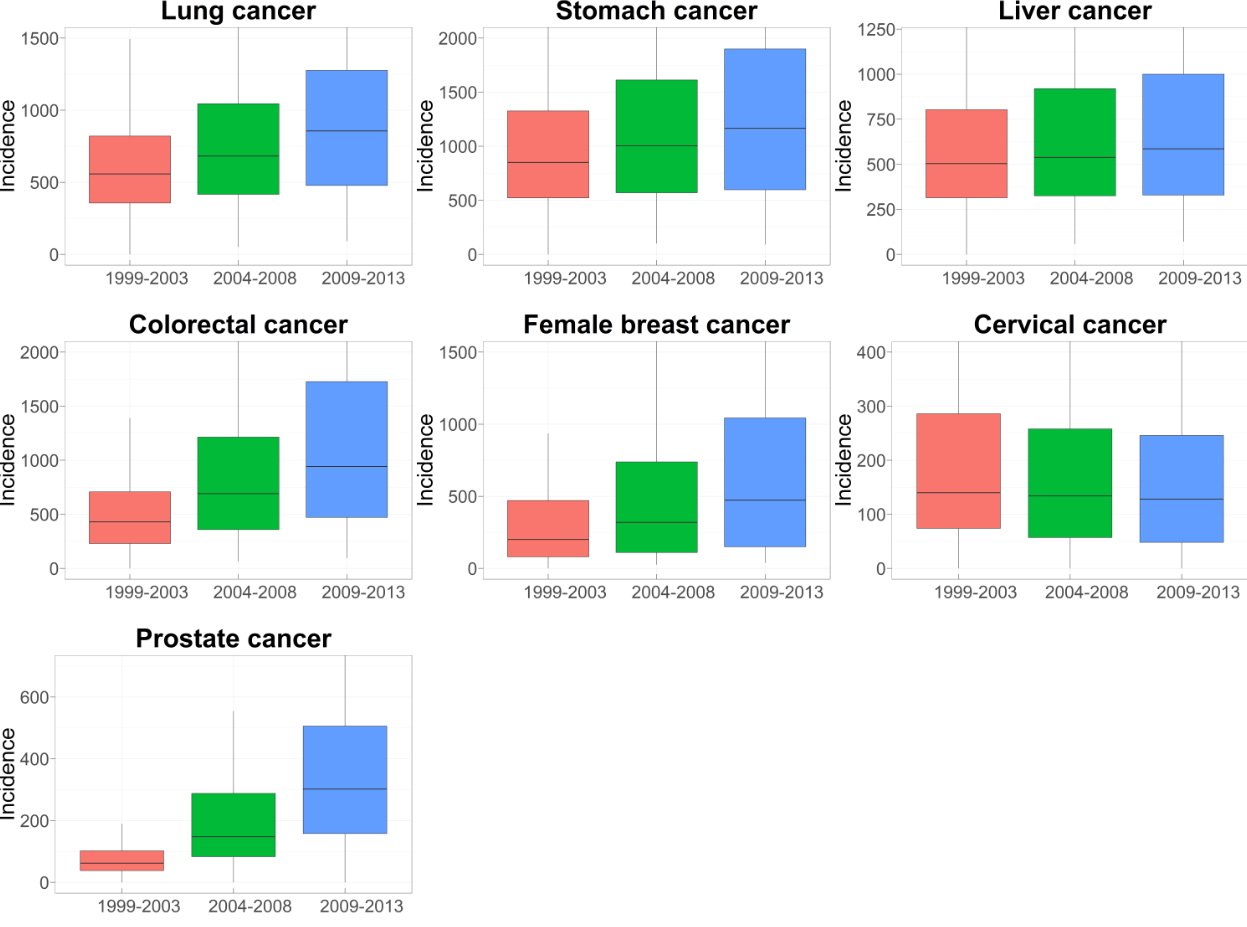


(B) Mortality


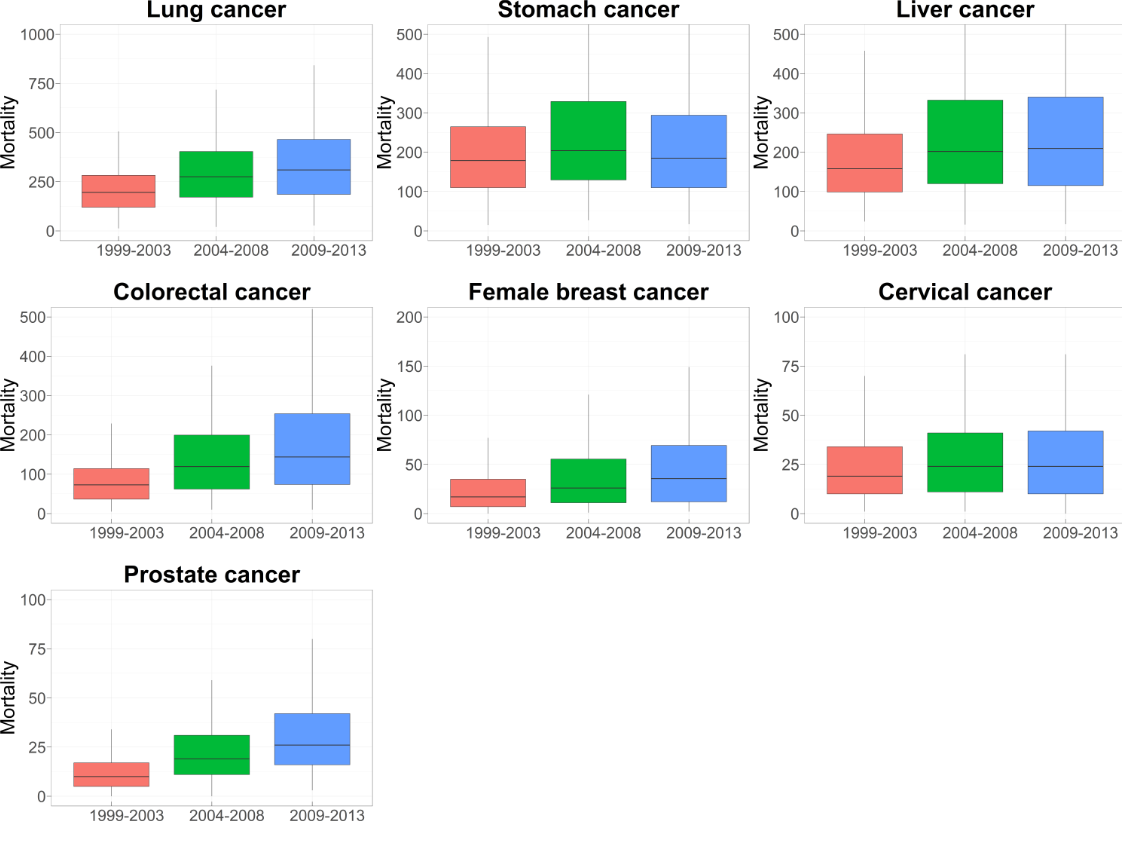


Figure S 2. Box plots of the numbers of new cases (A) and deaths (B) across 244 districts by three time periods and seven cancer types for 1999-2013 in South Korea

|  | 1999-2003 | 2004-2008 | 2009-2013 |
| --- | --- | --- | --- |
| Lung cancer | **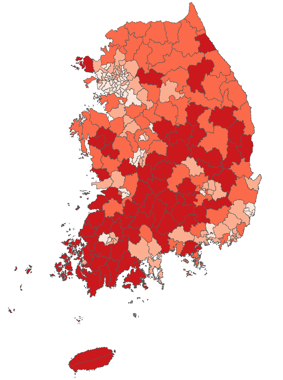** | **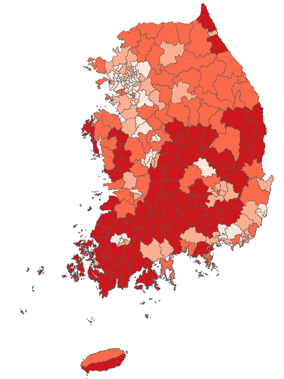** | **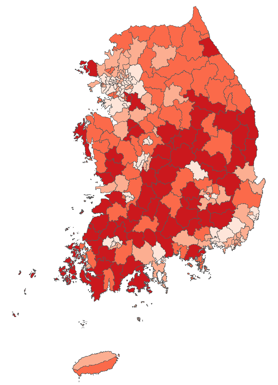** |
|  | **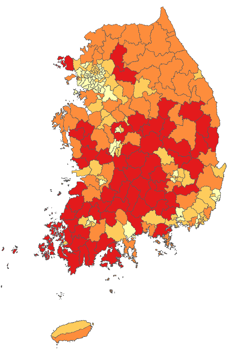** | **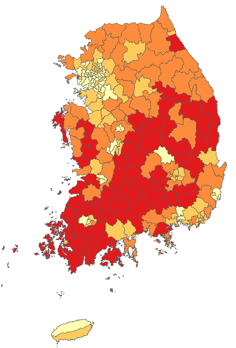** | **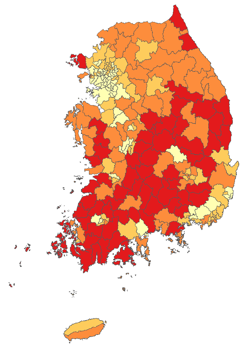** |
| Stomach cancer | **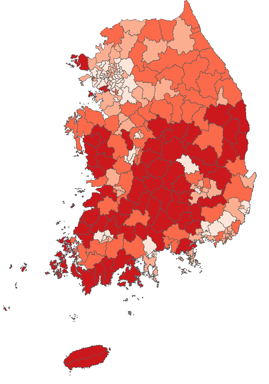** | **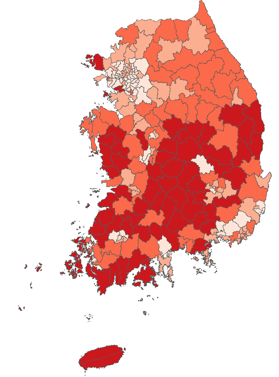** | **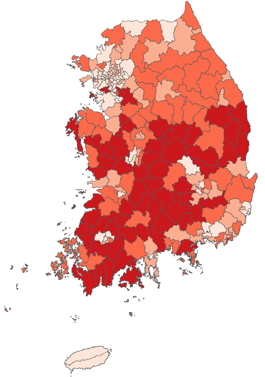** |
|  | **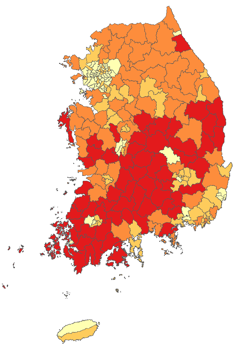** | **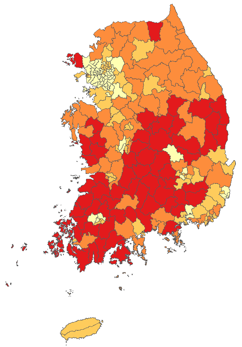** | **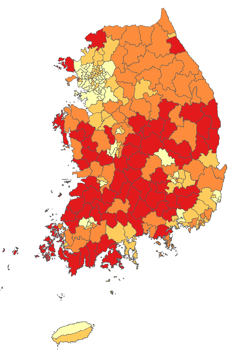** |
| Liver cancer | **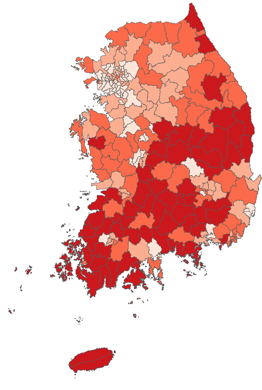** | **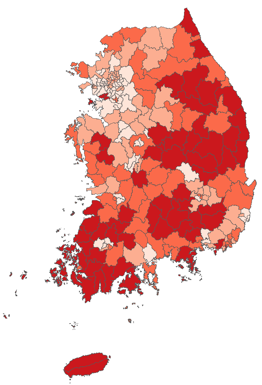** | **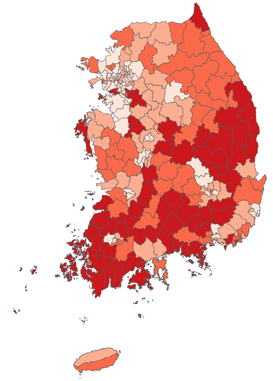** |
|  | **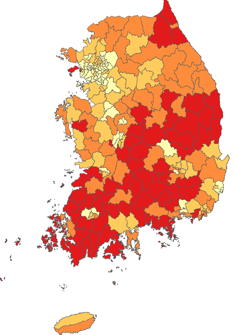** | **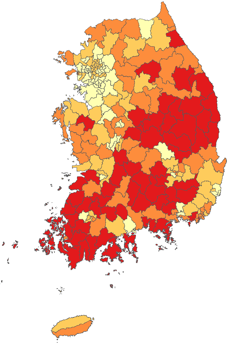** | **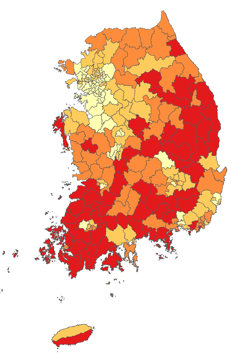** |
| Colorectal cancer | **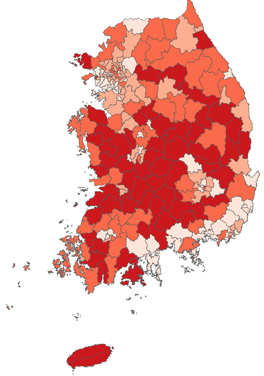** | **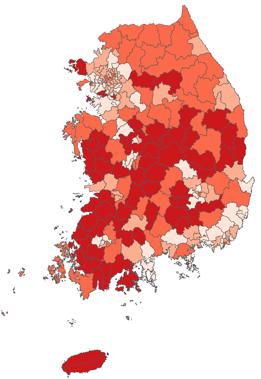** | **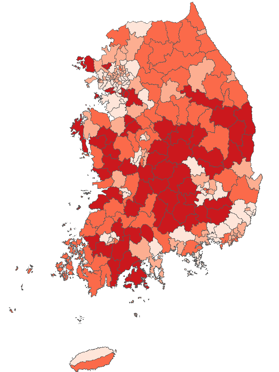** |
|  | **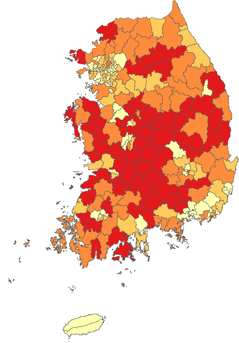** | **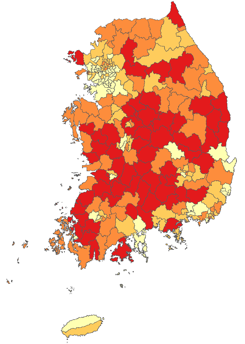** | **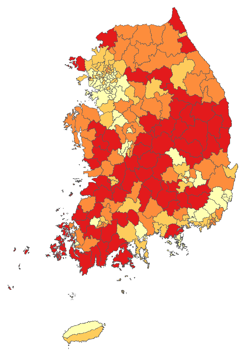** |
| Breast cancer | **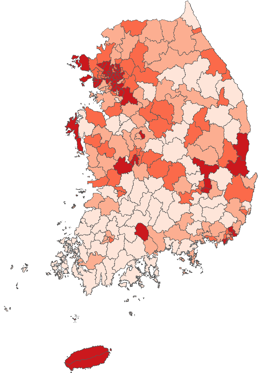** | **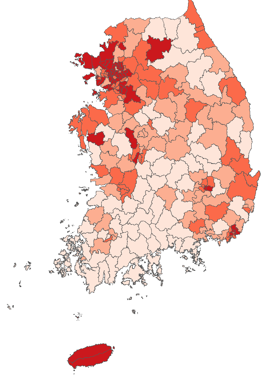** | **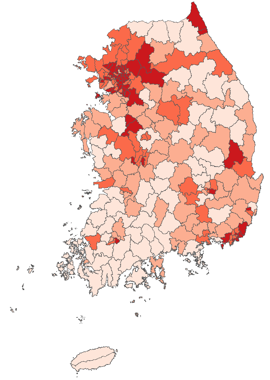** |
|  | **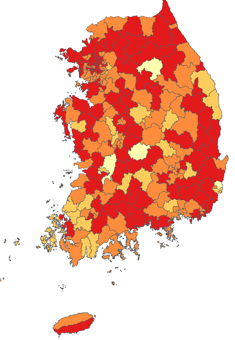** | **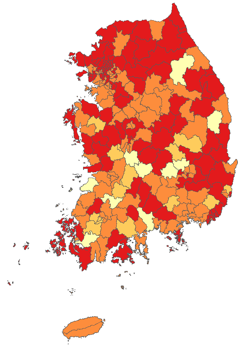** | **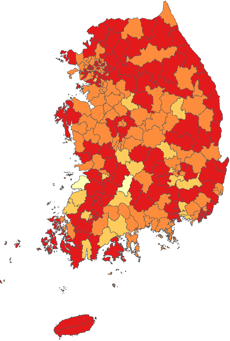** |
| Cervical cancer | **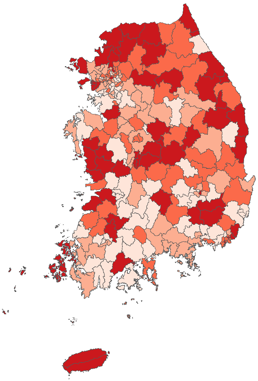** | **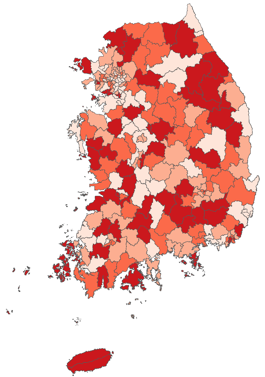** | **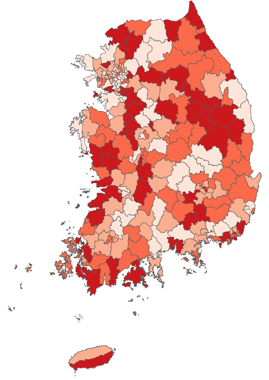** |
|  | **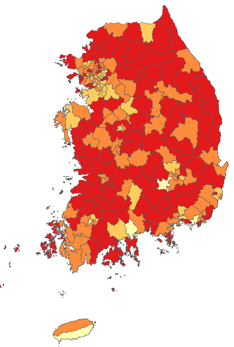** | **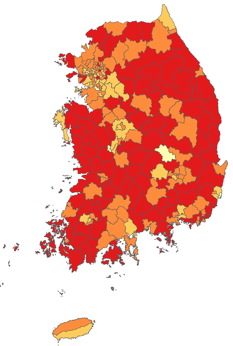** | **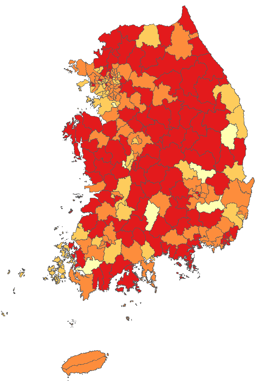** |
| 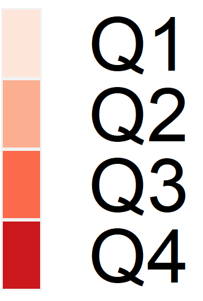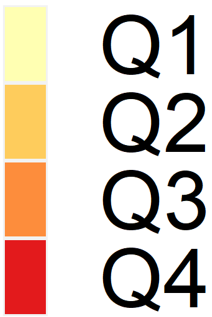Prostate cancer | **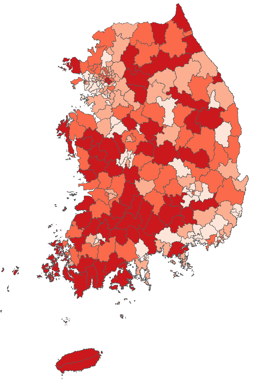** | **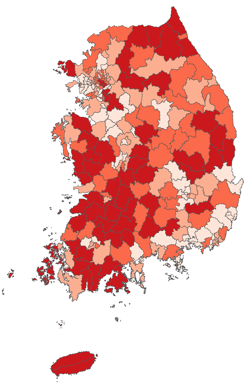** | **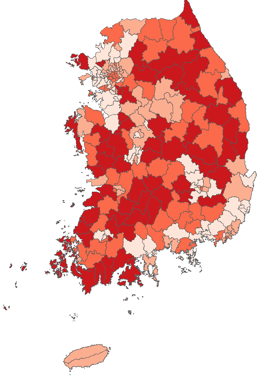** |
|  | **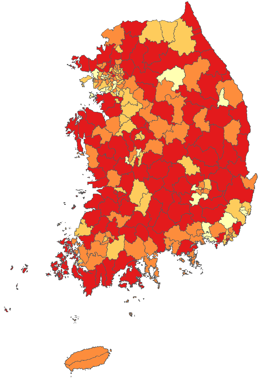** | **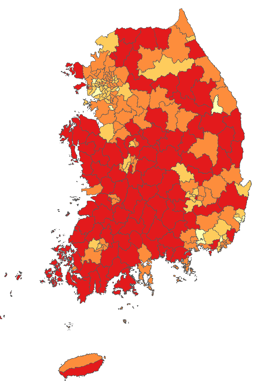** | **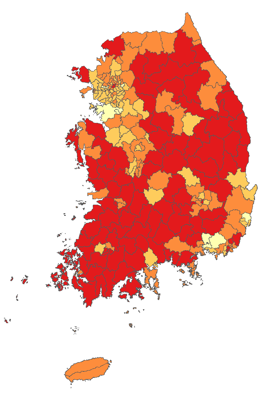** |

Figure S 3. Maps of crude incidence (above) and mortality (below) for seven cancers across 244 districts and three periods for 1999-2013 in South Korea

| Cluster change type | Period 1  (1999-2003) | Period 3  (2009-2013) | Cancer type  (Cluster region) | | | | | | | Possible explanations related to cancer control |
| --- | --- | --- | --- | --- | --- | --- | --- | --- | --- | --- |
|  |  |  | LU | ST | LI | CO | BR | CE | PR |  |
| 1 | High incidence | High incidence & mortality | SW | SW | SW |  |  |  |  | - High cancer incidence probably due to the increase of carcinogen exposure during the period 1 is followed by the increase in mortality during the period 3 after a lag period and the treatment intervention is not yet effective. |
| 2 |  |  |  | S | S |  |  |  |  | - Preventive and treatment interventions are not effective and do not change high incidence and mortality. |
| 3 |  |  |  |  |  | E |  |  | SW | - Cancer incidence and mortality both increase due to poor prevention as well as limited treatment advance. - Cancer incidence and mortality both are stable in the cluster region but decrease in other regions due to improved prevention intervention and treatment advance. |
| 4 |  |  |  |  |  | E | NW | NE | W | - Incidence increases as cancer screening or carcinogen exposure increased (1). - Cancer incidence is stable in the cluster region but decreases in other regions probably because of improved prevention. (2) |
| 5 | High mortality |  |  |  |  | C |  |  |  |  |
| 6 |  |  |  |  |  | W |  | NW | NW | - Effective preventive intervention such as decrease in carcinogen exposure result in incidence decrease. (3) |
| 7 |  | No cluster | SE | SE | S |  | SE |  | E | - Improved treatment advance leads to mortality decrease. (4) |
| 8 |  |  |  |  |  |  |  | C | SW | - Cancer treatment services deteriorated leading to relative increase in mortality. - Cancer mortality is stable in the cluster region but decreases in other regions possibly because of improved treatment advance. |

LU: lung; ST: stomach; LI: liver; CO: colorectal; BR: breast; CE: cervical; PR: prostate; C: central; E: east; NE: northeast; NW: northwest; S: south; SE: southeastern; SW: southwestern; W: west

Figure S 4. Change of incidence and mortality clusters over 15 years from 1999 through 2013 and possible explanation based on the nationwide cancer control program that has expanded since establishment in 1999 in South Korea

| Size = 5% (n = 12) | | | Size = 10% (n = 24) | | |
| --- | --- | --- | --- | --- | --- |
| 1999-2003 | 2004-2008 | 2009-2013 | 1999-2003 | 2004-2008 | 2009-2013 |
| Lung cancer | | | | | |
| 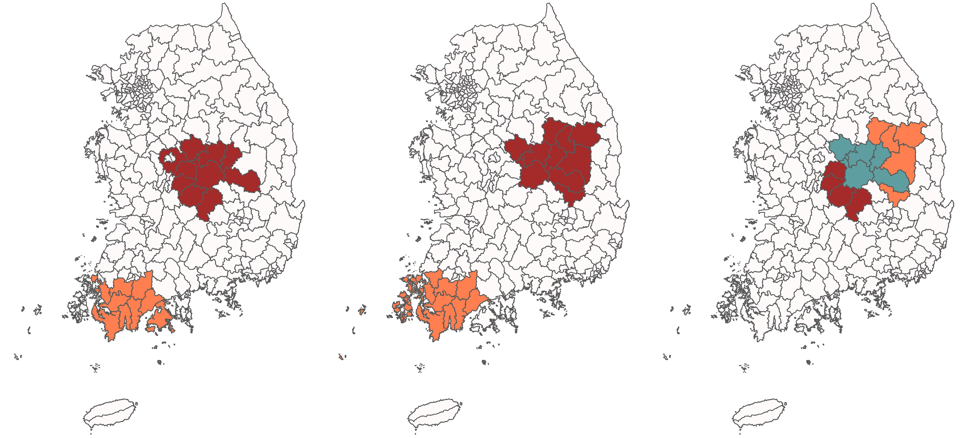 | | | 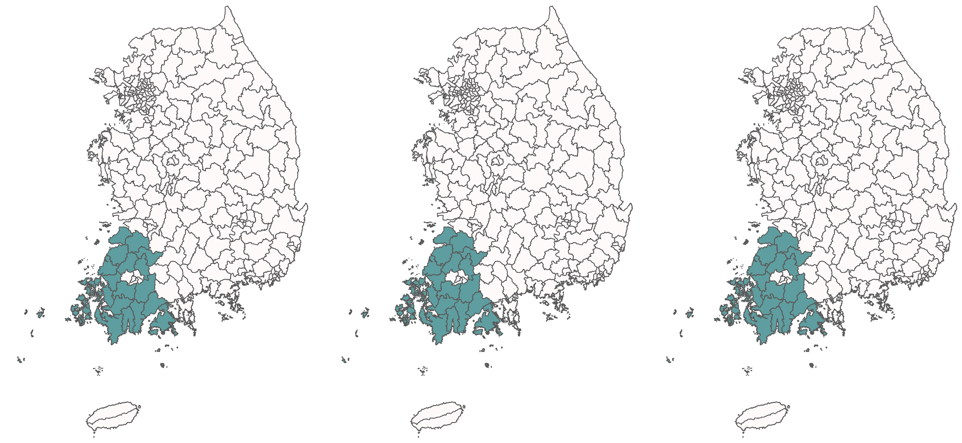 | | |
| Stomach cancer | | | | | |
| 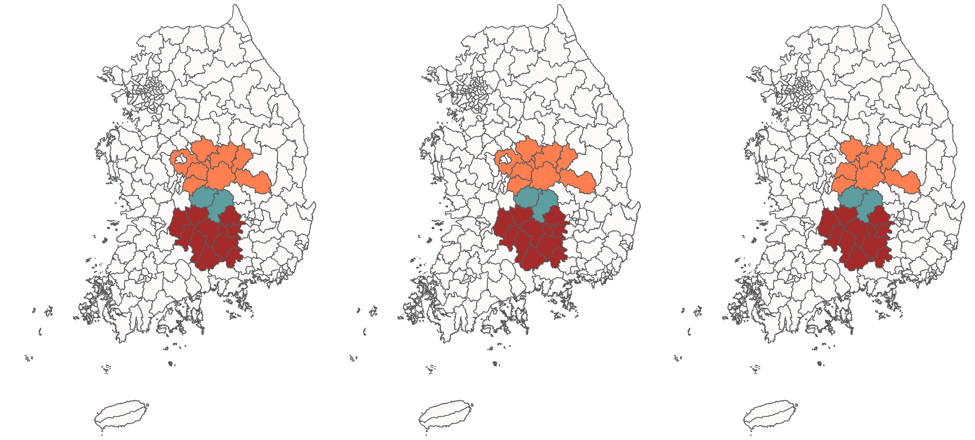 | | | 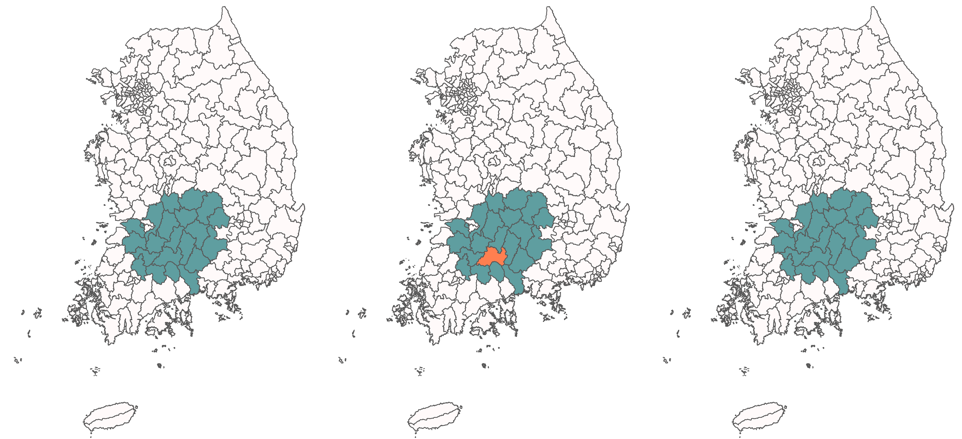 | | |
| Liver cancer | | | | | |
| 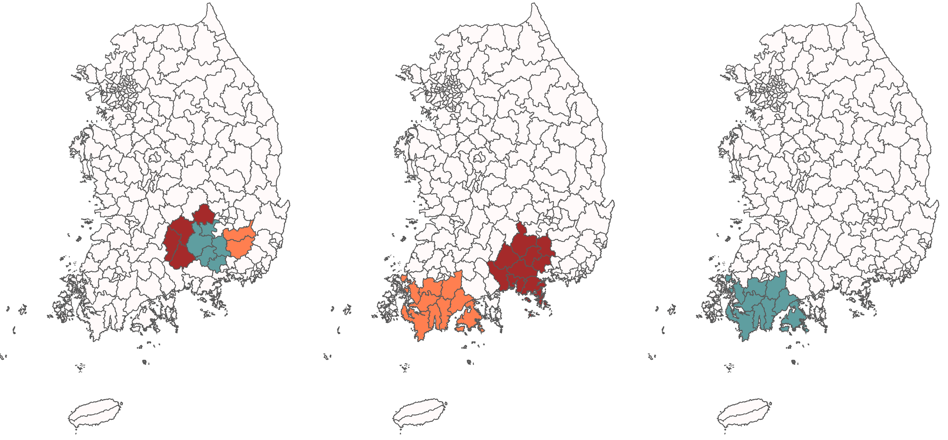 | | | 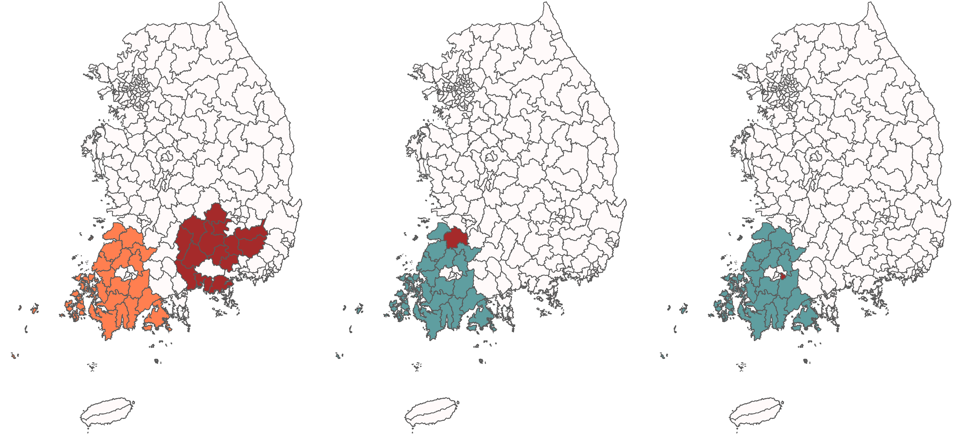 | | |
| Colorectal cancer | | | | | |
| 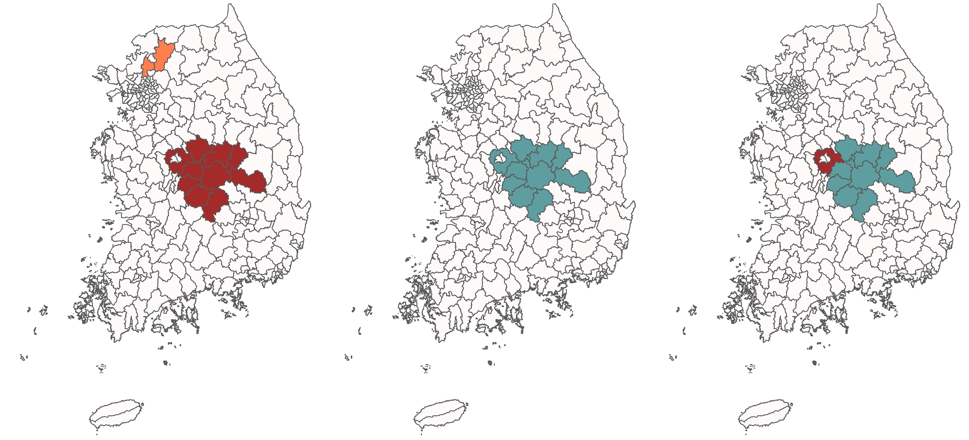 | | | 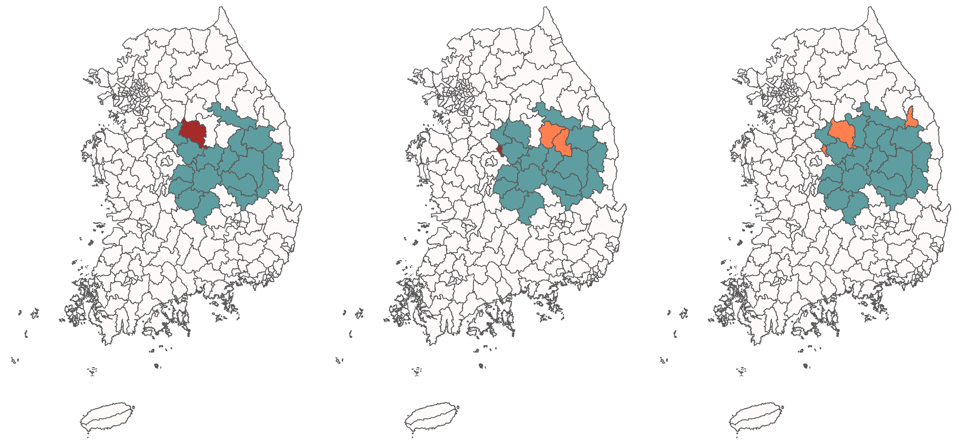 | | |
| Breast cancer | | | | | |
| 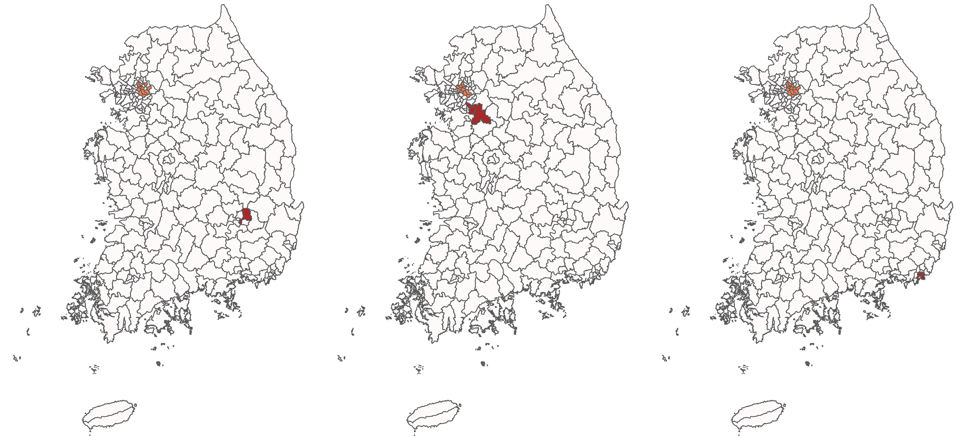 | | | 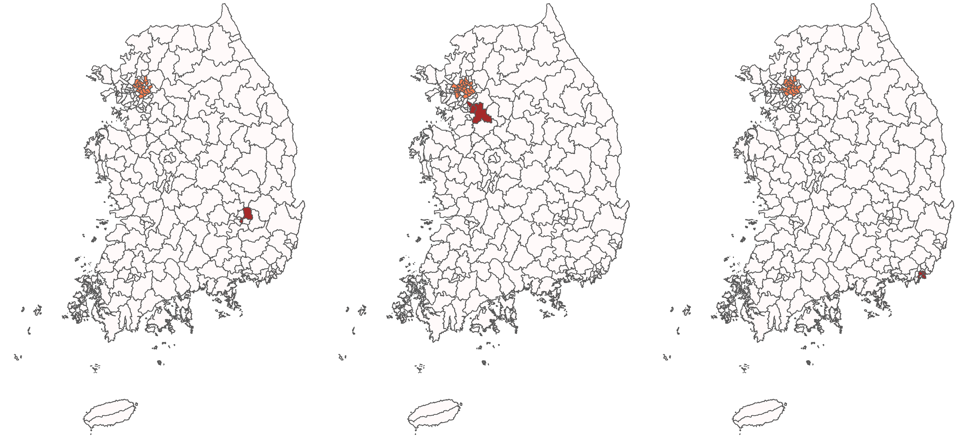 | | |
| Cervical cancer | | | | | |
| 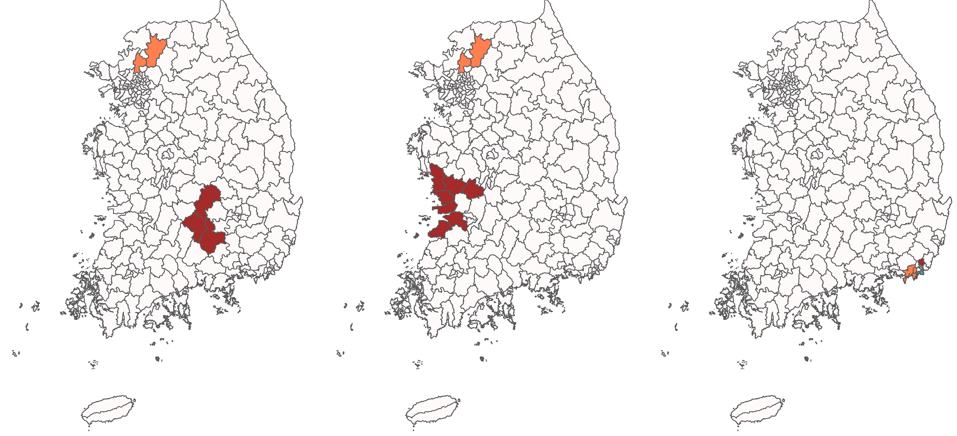 | | | 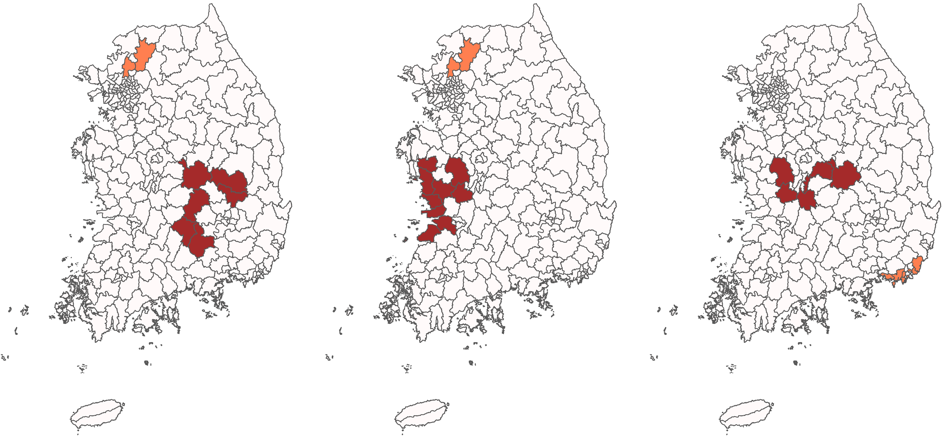 | | |
| Prostate cancer | | | | | |
| 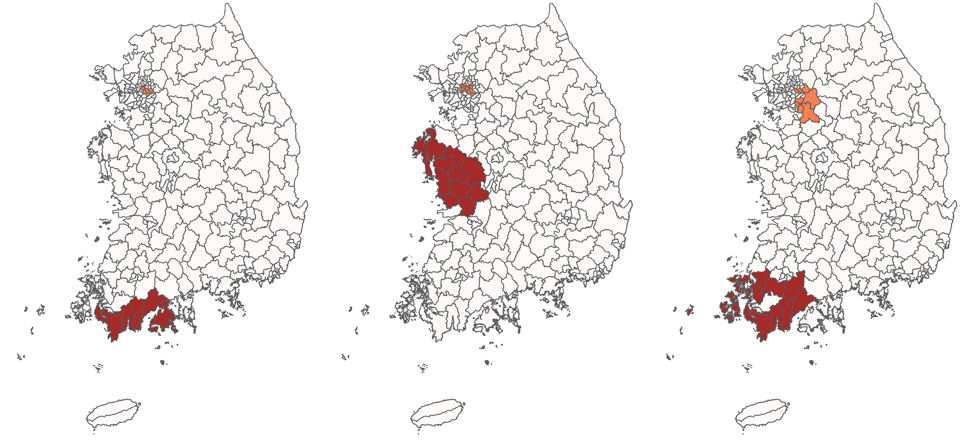 | | | 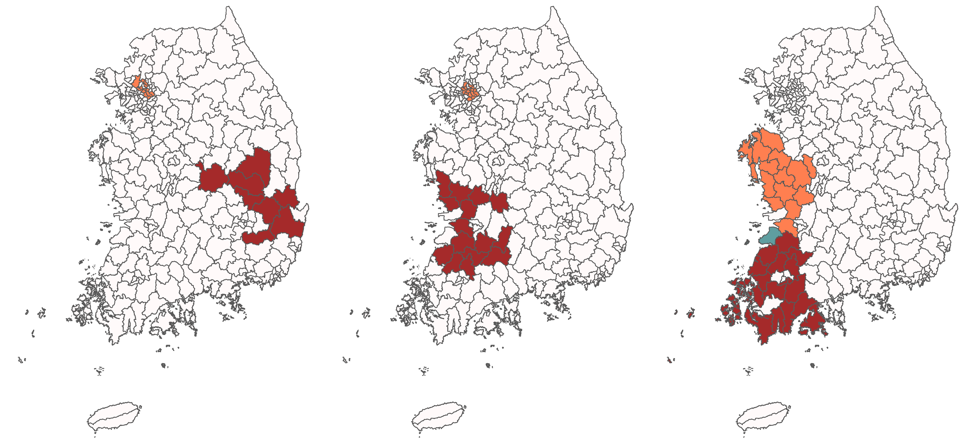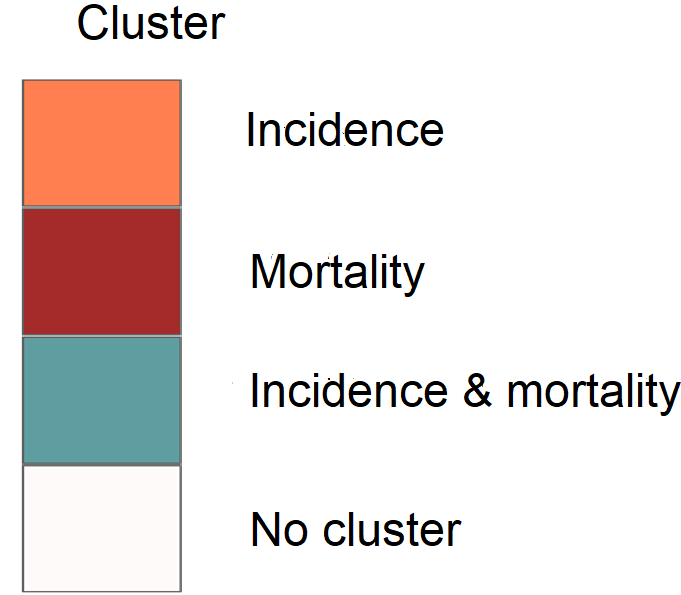 | | |

Figure S 5. Maps of primary clusters of cancer incidence and mortality by different scanning window size across 244 districts and three periods for 1999-2013 in South Korea

| Male | | | Female | | |
| --- | --- | --- | --- | --- | --- |
| 1999-2003 | 2004-2008 | 2009-2013 | 1999-2003 | 2004-2008 | 2009-2013 |
| Lung cancer | | | | | |
| 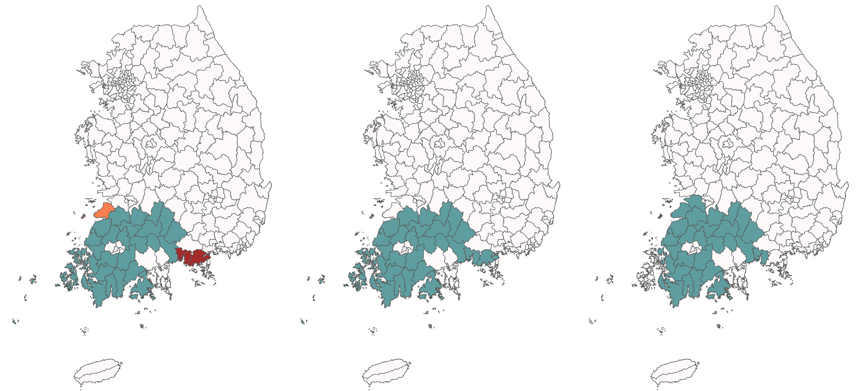 | | | 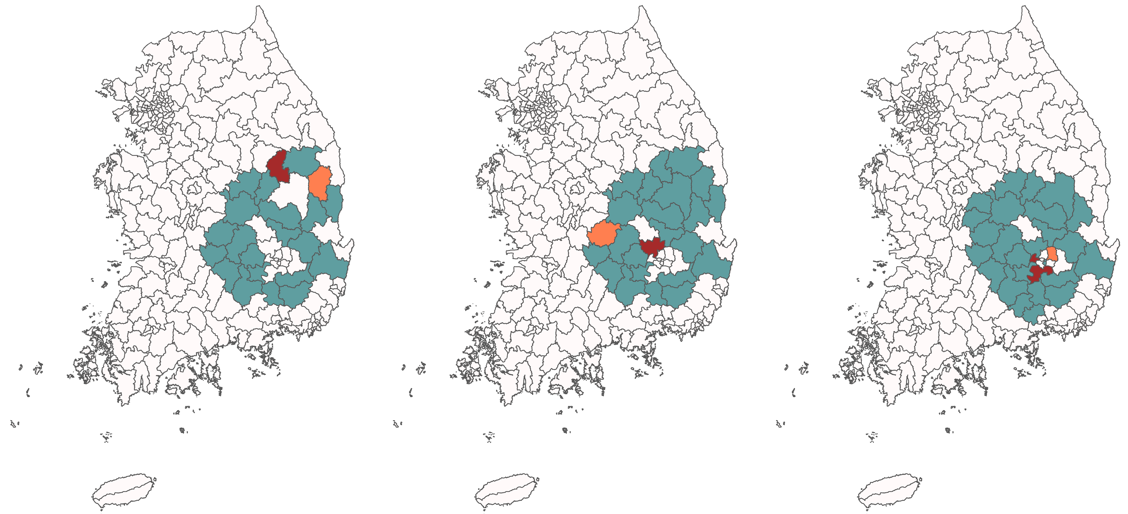 | | |
| Stomach cancer | | | | | |
| 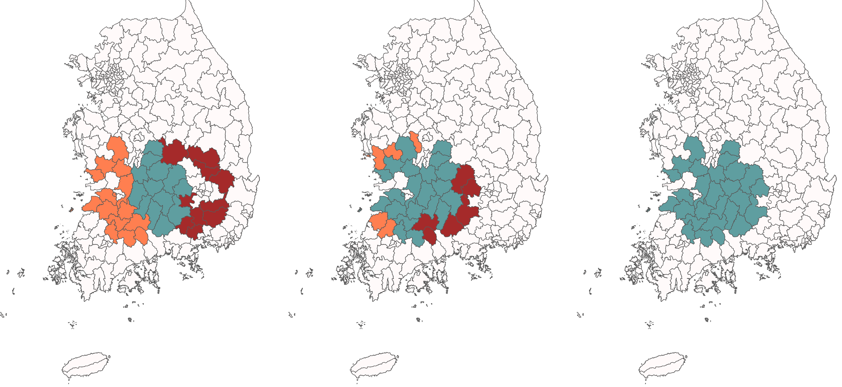 | | | 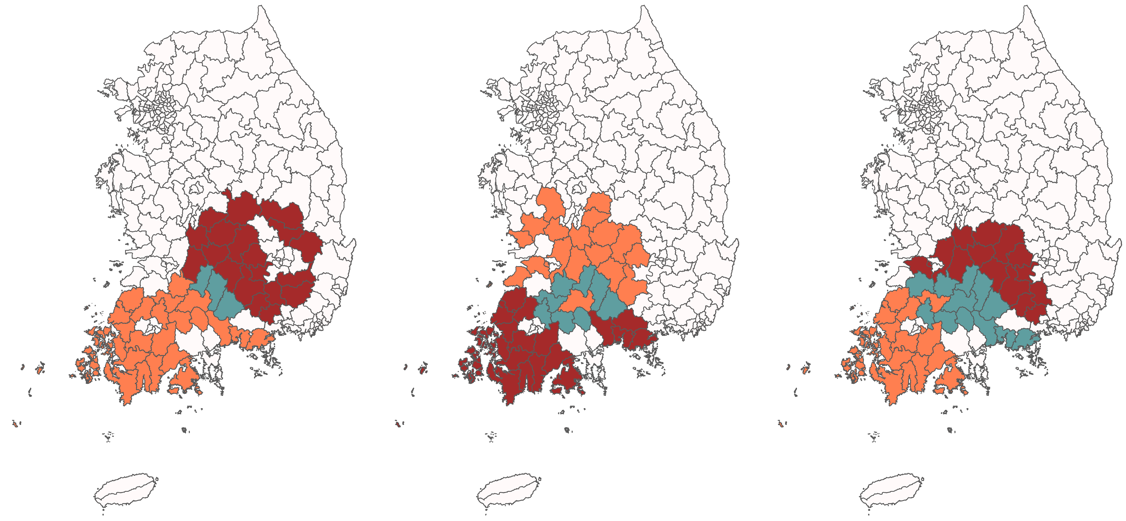 | | |
| Liver cancer | | | | | |
| 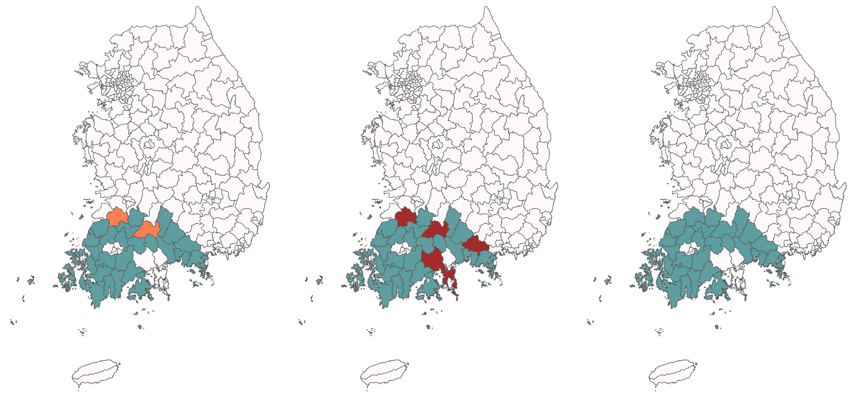 | | | 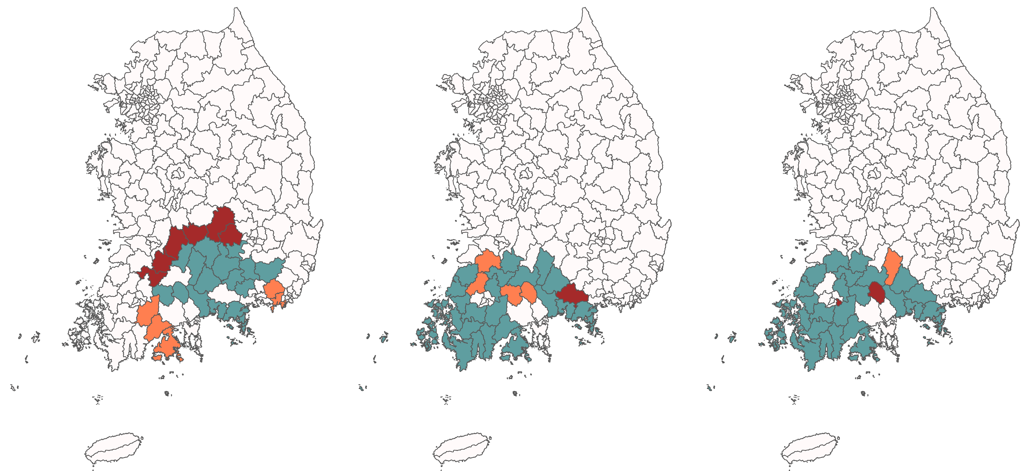 | | |
| Colorectal cancer | | | | | |
| 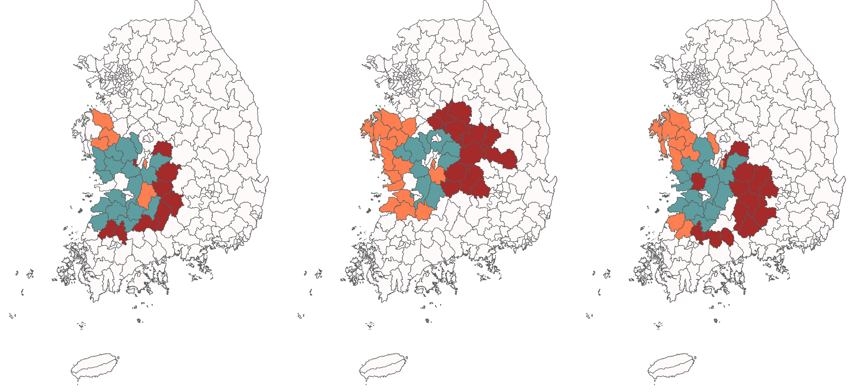 | | | 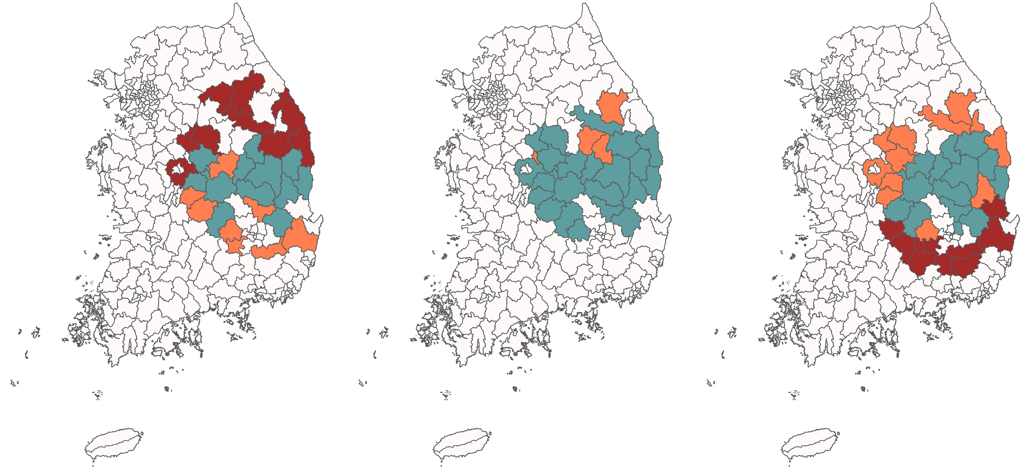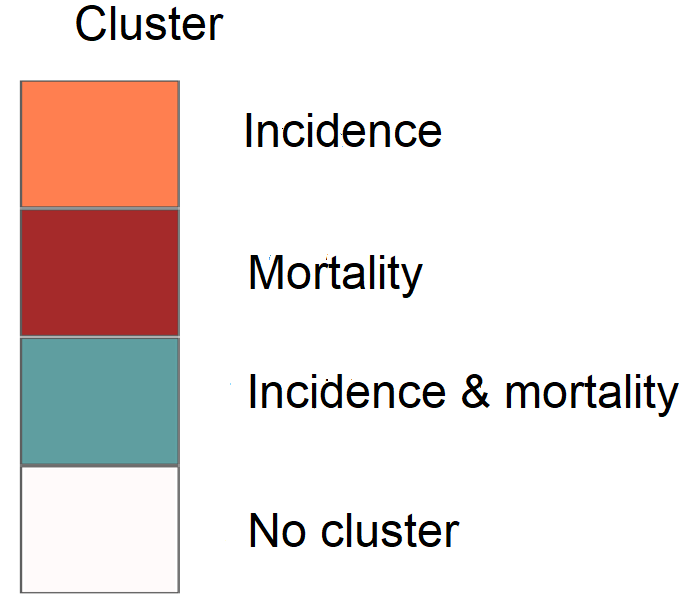 | | |

Figure S 6. Maps of primary clusters of cancer incidence and mortality by males and females across 244 districts and three periods for 1999-2013 in South Korea

| Cancer type | 1999-2003 (period 1) 2009-2013 (period 3) |
| --- | --- |
| Lung cancer | 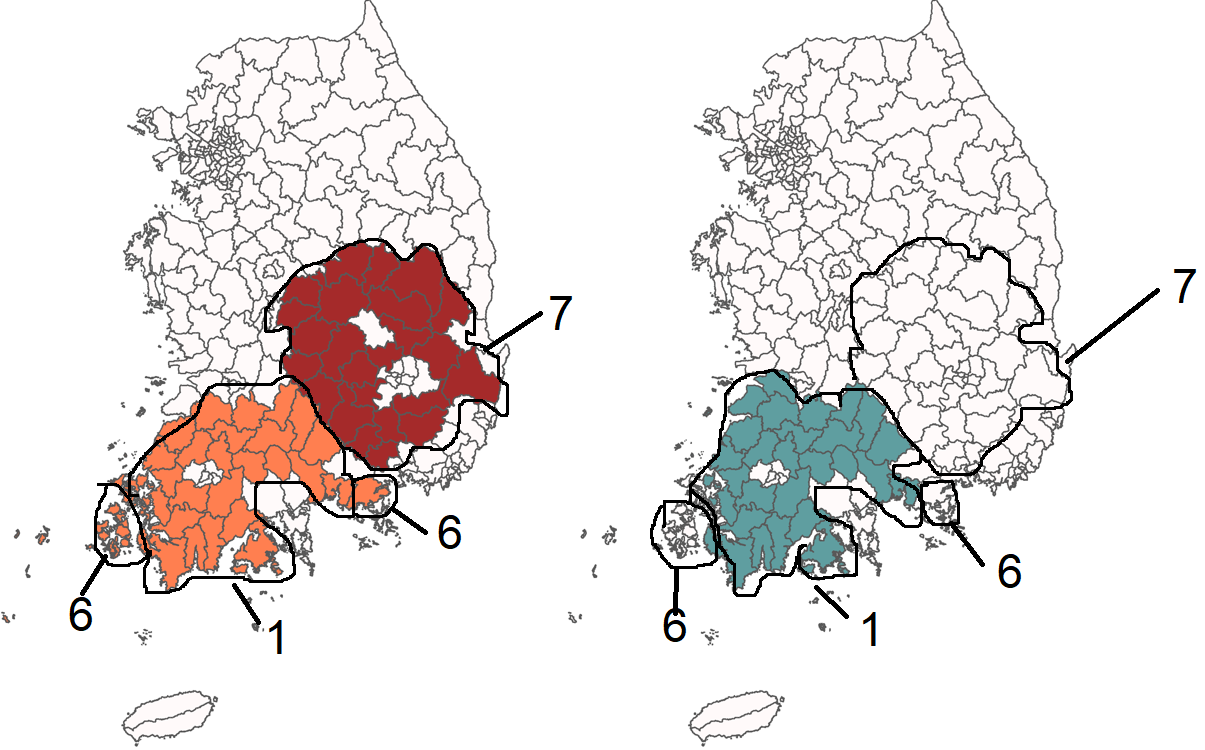 |
| Liver cancer | 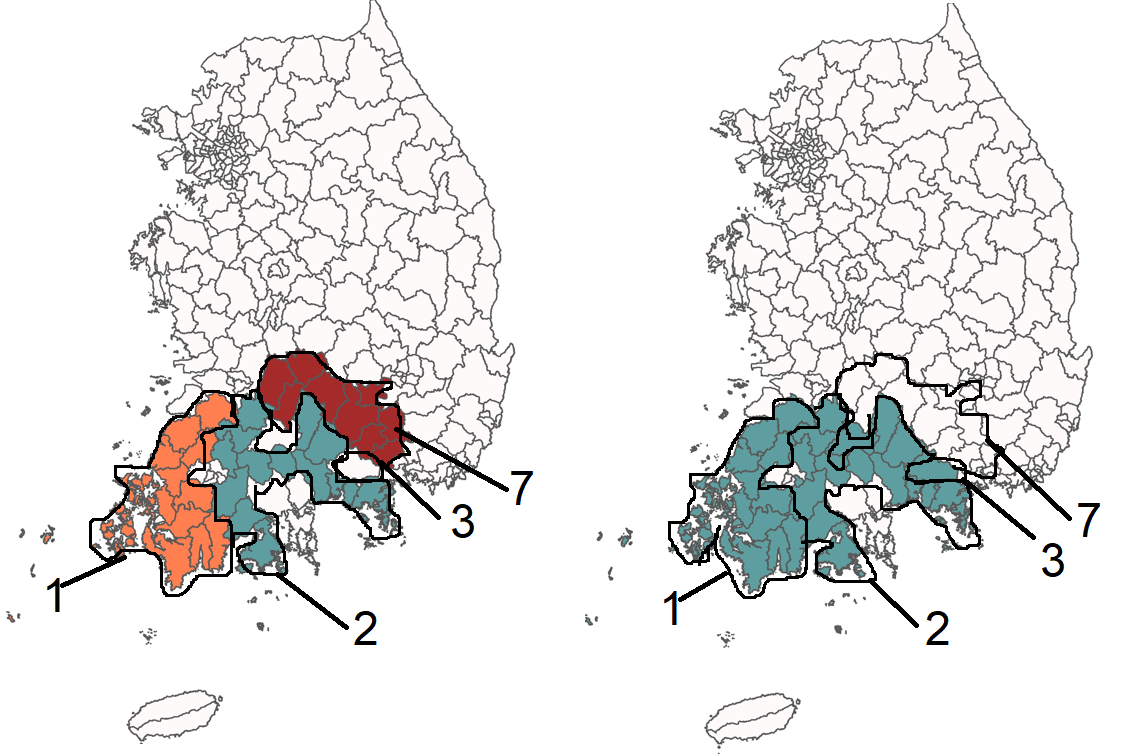 |
| Breast cancer | 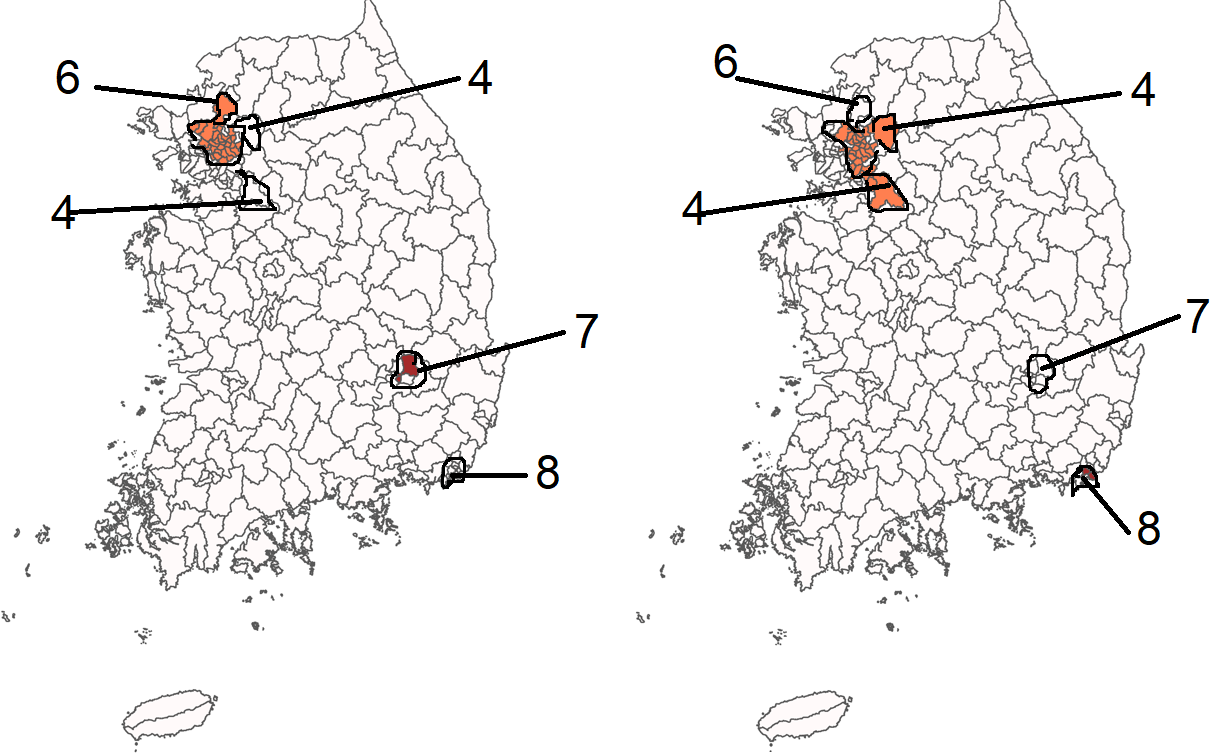 |
| Cervical cancer | 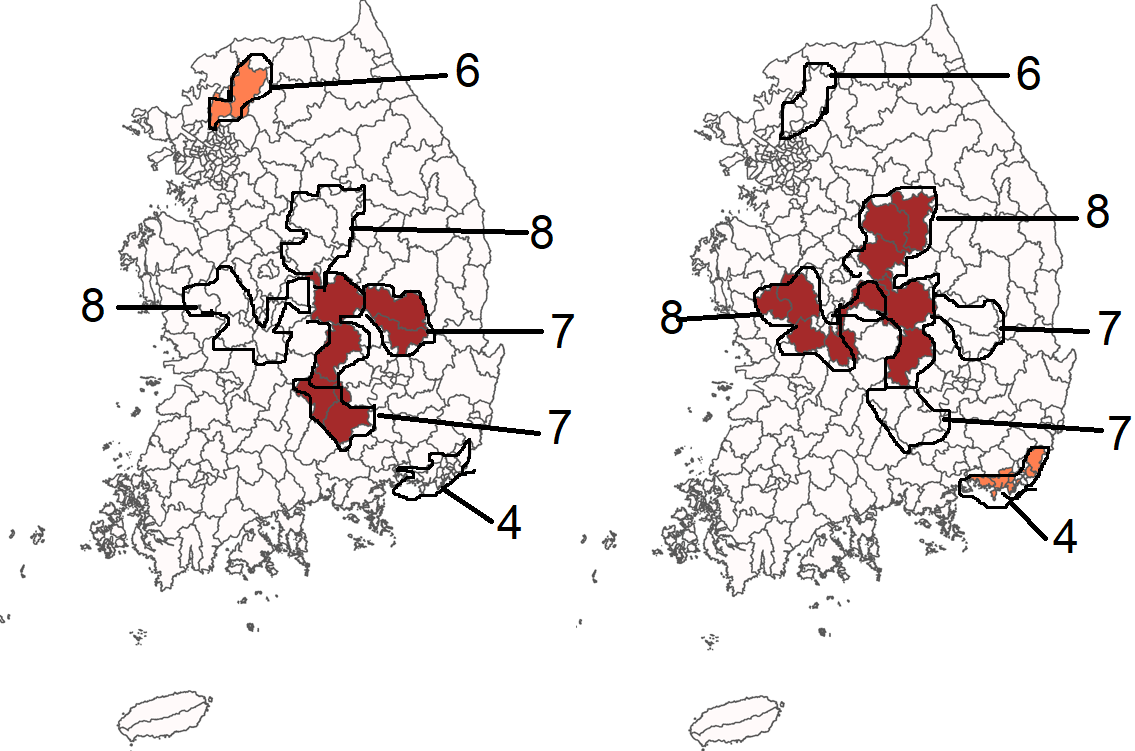 |
| Prostate cancer | 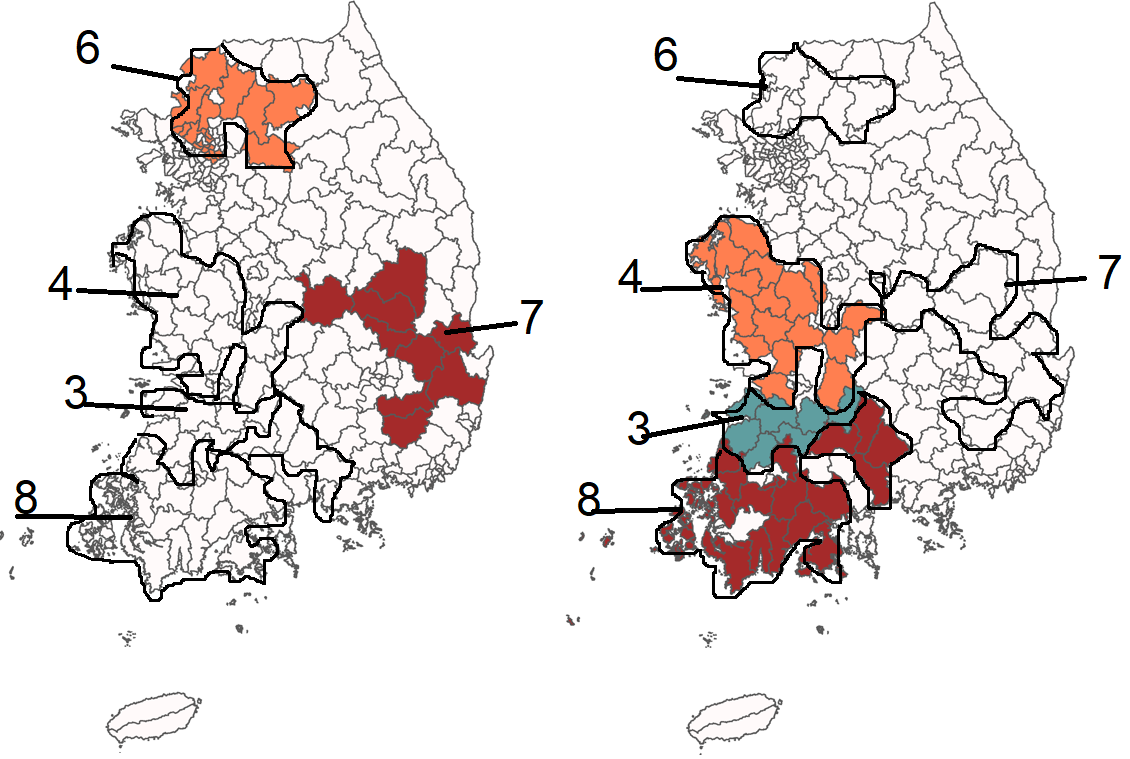 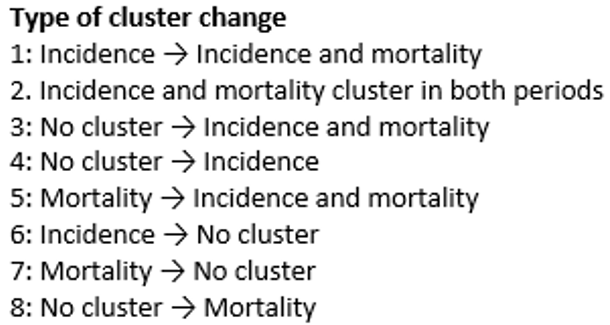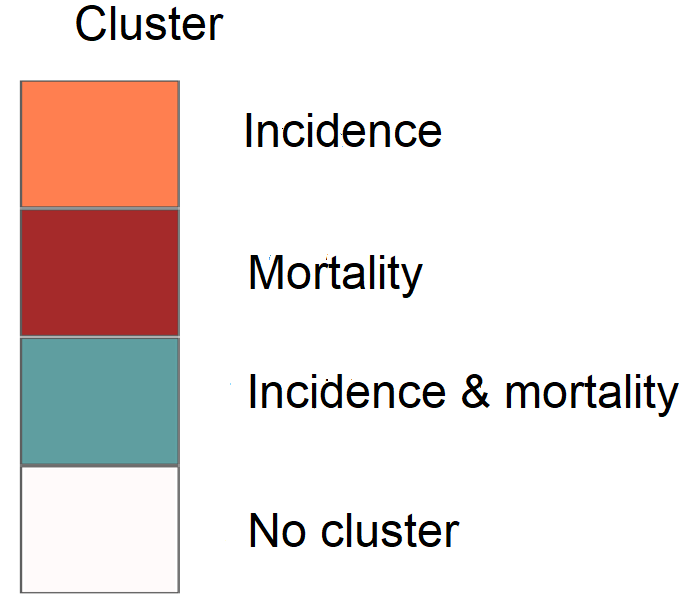 |

Figure S 7. Eight types of changes in cancer incidence and mortality clusters between the period 1 (1999-2003) and period 3 (2009-2013) by other cancer types in South Korea

**References**

1. Levin TR, Corley DA, Jensen CD, Schottinger JE, Quinn VP, Zauber AG, et al. Effects of Organized Colorectal Cancer Screening on Cancer Incidence and Mortality in a Large Community-Based Population. Gastroenterology. 2018;155(5):1383-91.e5.

2. Steinmaus C, Ferreccio C, Acevedo J, Yuan Y, Liaw J, Durán V, et al. Increased Lung and Bladder Cancer Incidence in Adults after In Utero and Early-Life Arsenic Exposure. Cancer Epidemiology, Biomarkers & Prevention. 2014;23(8):1529-38.

3. Funatogawa I, Funatogawa T, Yano E. Trends in smoking and lung cancer mortality in Japan, by birth cohort, 1949-2010. Bulletin of the World Health Organization. 2013;91(5):332-40.

4. Howlader N, Forjaz G, Mooradian MJ, Meza R, Kong CY, Cronin KA, et al. The Effect of Advances in Lung-Cancer Treatment on Population Mortality. New England Journal of Medicine. 2020;383(7):640-9.
